# Somatostatin neurons in prefrontal cortex initiate sleep-preparatory behavior and sleep via the preoptic and lateral hypothalamus

**DOI:** 10.1038/s41593-023-01430-4

**Published:** 2023-09-21

**Authors:** Kyoko Tossell, Xiao Yu, Panagiotis Giannos, Berta Anuncibay Soto, Mathieu Nollet, Raquel Yustos, Giulia Miracca, Mikal Vicente, Andawei Miao, Bryan Hsieh, Ying Ma, Alexei L. Vyssotski, Tim Constandinou, Nicholas P. Franks, William Wisden

**Affiliations:** 1 Department of Life Sciences, Imperial College London, London, UK; 2 Center for Excellence in Brain Science and Intelligence Technology, Chinese Academy of Sciences, Shanghai, China; 3 UK Dementia Research Institute, Imperial College London, London, UK; 4 Department of Electrical and Electronic Engineering, Imperial College London, London, UK; 5 Center for Neurotechnology, Imperial College London, London, UK; 6 Institute of Neuroinformatics, University of Zürich–ETH Zürich, Zürich, Switzerland; 7 Care Research and Technology Centre, UK Dementia Research Institute, London, UK

**Keywords:** Non-REM sleep, Physiology

## Abstract

The prefrontal cortex (PFC) enables mammals to respond to situations, including internal states, with appropriate actions. One such internal state could be ‘tiredness’. Here, using activity tagging in the mouse PFC, we identified particularly excitable, fast-spiking, somatostatin-expressing, γ-aminobutyric acid (GABA) (PFC^*Sst*-GABA^) cells that responded to sleep deprivation. These cells projected to the lateral preoptic (LPO) hypothalamus and the lateral hypothalamus (LH). Stimulating PFC^*Sst*-GABA^ terminals in the LPO hypothalamus caused sleep-preparatory behavior (nesting, elevated theta power and elevated temperature), and stimulating PFC^*Sst*-GABA^ terminals in the LH mimicked recovery sleep (non-rapid eye-movement sleep with higher delta power and lower body temperature). PFC^*Sst*-GABA^ terminals had enhanced activity during nesting and sleep, inducing inhibitory postsynaptic currents on diverse cells in the LPO hypothalamus and the LH. The PFC also might feature in deciding sleep location in the absence of excessive fatigue. These findings suggest that the PFC instructs the hypothalamus to ensure that optimal sleep takes place in a suitable place.

## Abstract (web summary)

The sleep drive builds with time spent awake. Tossell et al. show in mice that tiredness triggers neurons in the prefrontal cortex to instruct the hypothalamus to initiate nesting and then sleep itself, ensuring that sleep occurs in a safe place.

## Main

Animals and humans undertake specific behaviors as they become drowsy^1–8^. In the case of mice, the closer in time they are to sleeping, the more likely it is that nest building occurs^7^. Nesting and bedding in general serve as a protective environment during sleep and provide a thermal microclimate that promotes skin warming that, in turn, induces NREM sleep and body cooling mediated by circuitry in the medial preoptic (MPO) hypothalamus^3,9^. The lower body and brain temperatures in NREM sleep might be needed for sleep’s function^3,10,11^, because the same MPO neurons that lower body temperature also induce NREM sleep^9^. Preventing nesting induces insomnia (sleep–wake fragmentation)^7^.

Compared with the extensive web of sleep–wake-regulating circuitry^12,13^, we know only fragments of the circuitry that influences nesting. During spontaneous nesting that occurs before sleep, electroencephalography (EEG) theta power, particularly at 7 Hz, is elevated^7^. Nest building before sleep can be initiated by inhibiting dopamine neurons in the ventral tegmental area (VTA) and stimulating glutamatergic neurons in the LH^1,7^; both types of cells are widely projecting, but the relevant targets for nesting are uncharacterized.

We wondered whether the neocortex exerts any top–down influence on sleep-preparatory behavior (nesting) and coordination with sleep. The neocortex does seem to contribute to sleep regulation directly. Neocortical *Sst*-expressing GABA cells enhance NREM-like sleep and slow-wave activity by an unknown mechanism^14,15^, and genetic silencing of layer V pyramidal and hippocampal dentate granule neurons blocks the characteristic increase in EEG delta power of recovery sleep (RS) following sleep deprivation (SD) and increases the amount of wakefulness during the active (‘lights-off’) period of mice^16^. The characteristic increase in delta power of NREM RS reflects a deeper sleep^17^ and is part of the sleep homeostatic model. In awake and behaving animals, local delta NREM-like oscillations develop in different regions of the neocortex following use-dependent activity^18–22^, and this increase in local delta power depends on increasing chloride concentrations inside pyramidal neurons as wakefulness progresses^23^.

Excitability throughout the neocortex increases with time spent awake^24^. During SD and RS, particular types of neocortical GABAergic neurons, such as nitric oxide synthase 1 (*Nos1*)-expressing neurons, become active^25–27^. But the PFC seems particularly sensitive to SD^28–31^, which causes functional connectivity to degrade in the PFC more than in other neocortical areas^29,30^; indeed, there is a selective buildup of glutamate and glutamine in the PFC relative to the VC during daylong cognitive work, which could signal tiredness^31,32^. Nearly a third of the neurons in the monkey PFC increase their firing rate during cognitive disengagement (for example, on becoming drowsy), eye closure and sleep^33,34^.

Conceptually, the PFC stores and creates combinations of purposeful actions^35^, implementing survival and autonomic processes, such as defensive responses^36^, and a selection of behavioral states in response to challenges^37^. Given the heightened sensitivity of the PFC to SD, we therefore hypothesized that the PFC could potentially link sleep pressure, which builds up as wakefulness increases (that is, during SD)^17^, with sleep-preparatory behaviors such as nesting and with sleep itself. We find that, when mice are deprived of sleep, somatostatin (SST)-expressing GABA cells in the PFC (PFC^*Sst*-GABA^ cells) induce subsequent nesting, elevated theta power and body temperature increase through projections to the LPO hypothalamus while nest building is taking place. Additionally, through projections to the LH, PFC^*Sst*-GABA^ cells induce NREM RS with elevated delta power and an associated body temperature decrease. This combined PFC–hypothalamic circuitry could ensure that, if an animal is tired, sleep takes place in a safe environment that promotes RS.

## Results

### Tagging PFC^GABA^ cells during SD, nesting and RS

**Extended Data Fig. 1:**
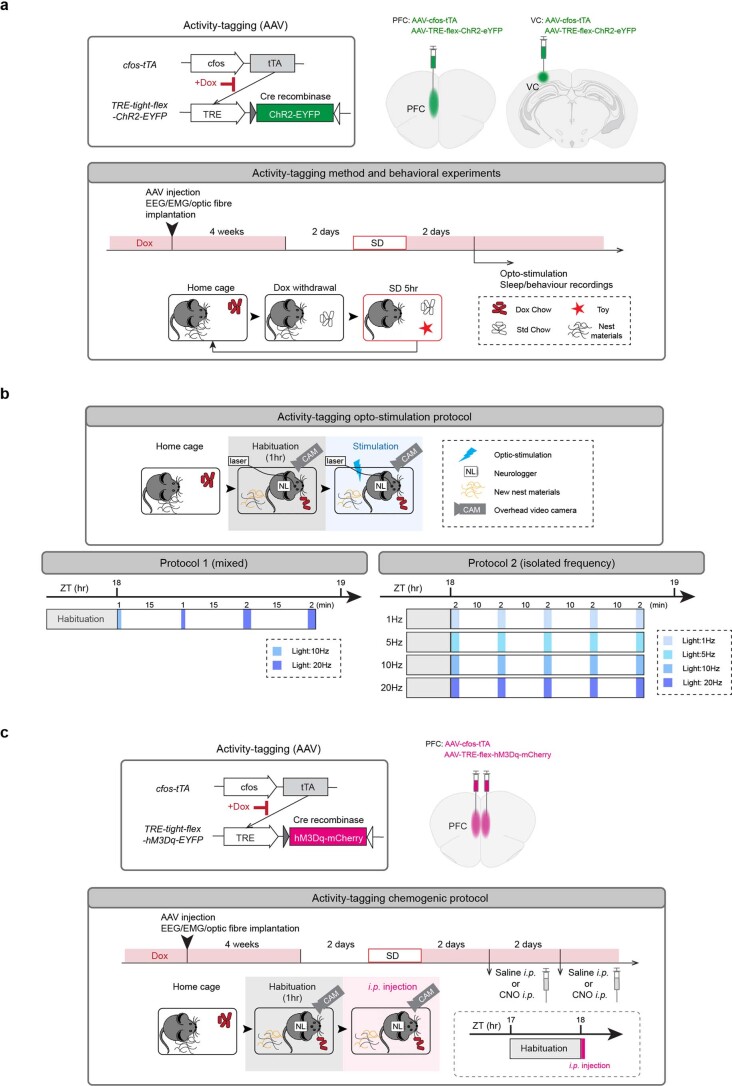
Experimental schematics for activity-tagging (supports Figs. 1–4). **a**, Schematic of activity-tagging method and behavioral experiments for *Vgat*-expressing neurons in the PFC with ChR2-EYFP during sleep deprivation (SD). Dox chow was re-introduced immediately after 5hrs of SD (ZT0-ZT5) and animals were allowed to carry out post-SD nesting and RS without any disturbance while the activity-tagging system is gradually repressed. **b**, Schematic of activity-tagging opto-stimulation protocols. Prior to habituation period (1 hr), nesting materials in the home cage was reduced and mixed with new nest materials (total approx. 8 g) and food pellets were moved to the far end away from the nesting materials and closer to the water bottle spout. Two separate opto-stimulation protocols were given at ZT18 after 1 hour of habituation. **c**, Schematic of activity-tagging chemogenic reactivation protocols. Saline and CNO were given 2 days after activity-tagging via *i.p*. injection with blinding. The mice had 2 days rest time between the 1^st^ and 2^nd^ injections.

To investigate GABAergic cells that became active following SD, nesting and RS in the PFC (prelimbic, infralimbic and medial orbital subdivisions^35^) and, as a comparison, in the visual cortex (VC), we used c-Fos-based activity tagging^9,38,39^. Tagging was restricted to cells expressing the *Slc32a1* (*Vgat*) gene in the PFC or the VC (*Vgat*-PFC-ChR2-Tag and *Vgat*-VC-Channelrhodopsin-2 (ChR2)-Tag mice, respectively) (see Extended Data Fig. 1a and Supplementary Table 1 for a summary of the method and a list of mouse lines). For tagging, mice were deprived of sleep for 5 h by presenting them with new objects at ZT 0 (start of the light period, greatest sleep-propensity period; ZT, zeitgeber time (hours)). Mice were then placed back in their home cages with doxycycline (Dox)-containing chow (gradually repressing the activity-tagging system) (Extended Data Fig. 1a).

**Fig. 1:**
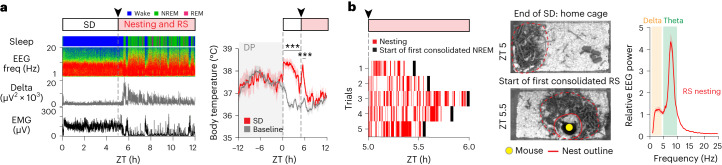
SD, nesting behavior, RS and corresponding changes in core body temperature. **a**, Example EEG–EMG traces and sleep stage state and mean core body temperature during SD and RS and post-SD nesting activity and nest materials in the home cage. *N* = 7 *Vgat*^Cre^ mice, baseline versus SD, *P* = 4.78 × 10^−4^ (ZT 0–5), *P* = 1.12 × 10^−3^ (ZT 5–6) with two-way repeated-measures (RM) ANOVA with Bonferroni correction. Freq, frequency. **b**, Raster plot of RS nesting, example nest image in the home cage and relative EEG spectrum. Red raster and solid black bars indicate nesting and onset of first consolidated NREM RS. Yellow dot, position of mouse; dashed red line, outline of nesting materials; solid red line, outline of nest. *N*, number of biologically independent mice. ***P < 0.001. Mean (line) ± s.e.m. (shading). See also Extended Data Figs. 1 and 2. DP, dark period.

**Extended Data Fig. 2:**
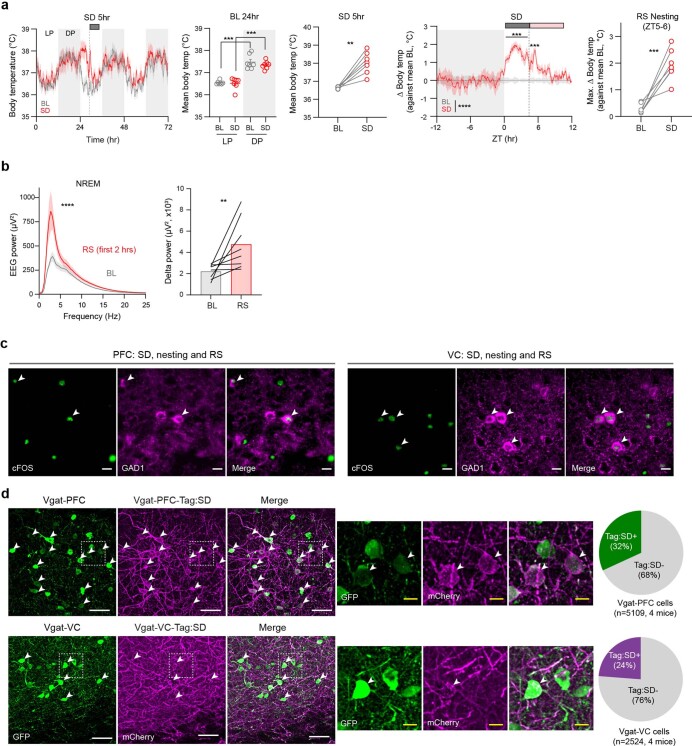
Activity-tagging of GABA neurons in the prefrontal cortex (PFC) and visual cortex (VC) (supports Figs. 1, 2). **a**, Core body temperature changes during 5 hrs of SD (ZT0-5) and subsequent RS (top panels). *Left*: Mean body temperature increased in the dark period by 1 °C in baseline (BL) 24 hrs (LP vs. DP, BL: *P* = 0.0006, SD: *P* = 0.0006, Two-sided Mann-Whitney *U* test). *Middle*: Mean body temperature during SD compared with the BL (*P* = 0.00101, Two-tailed paired *t*-test). *Top 2*^*nd*^ *right*: Time course of Δ body temperature against mean BL temperature ((ZT0-5): *P* = 5.20 × 10^−4^, (ZT5-6): *P* = 9.47 × 10^−4^, (ZT0-6, interaction): *P* = 5.79 × 10^−18^, 2-way RM ANOVA with Bonferroni correction). *Far right*: max. Δ body temperature in ZT5-6 (*P* = 0.00076, Two-tailed paired *t*-test). **b**, EEG power spectrum of NREM sleep in the first 2 hours of RS (*P* = 2.29 × 10^−33^, 2-way RM ANOVA with Bonferroni correction) and mean Delta power (*P* = 0.0068, Two-tailed Wilcoxon matched-pairs signed rank test). *Vgat*-Cre mice, *n*_*mice*_ = 8. **c**, Representative images of endogenous cFOS protein (green) colocalized with GAD1 protein (magenta) in the PFC and VC following SD/nesting/RS. **d**, Percentage of GABAergic tagged neurons in the PFC and VC as assessed by hM3Dq-mCherry expression (magenta). Non-tagged GABA neurons are defined by EYFP expression (green) after co-injecting *AAV-flex-EYFP* into the PFC and VC of *Vgat-Cre* mice. *Vgat-PFC-YFP::Vgat-PFC-hM3Dq-Tag:SD* mice; *n*_*neuron*_ = 5109, *N*_*mice*_ = 4, *Vgat-VC-YFP::Vgat-VC-hM3Dq-Tag:SD* mice; *n*_*neuron*_ = 2524, *N*_*mice*_ = 4. Mean (line) ± SEM (shading) in **a, b**. Arrowheads in **c**, **d** indicate co-expression. *n*_*neuron*_ = number of neurons, *N*_*mice*_ = number of biologically independent mice. **P < 0.01, *** *P* < 0.001 **** *P* < 0.0001. LP: light period, DP: dark period. Scale bars: 25 µm (**c**), 50 µm (**d**, white), 10 µm (**d**, yellow).

Before SD, body temperature oscillated diurnally over 24 h (Extended Data Fig. 2a)^9,40^, with a lower body temperature (by about 1 °C) during the ‘lights-on’ (sleep time) period. During SD, the body temperature increased by about 2 °C but declined partially during the later part of SD (Fig. 1a and Extended Data Fig. 2a). After SD, mice either preferentially went to a prebuilt nest in their home cage and improved it or, if there was no nest, they built one before starting their RS (Fig. 1b). During the nesting activity, EEG showed a peak in theta frequencies, as found previously^7^, and body temperature was elevated (Fig. 1a,b and Extended Data Fig. 2a). After nesting, RS had an increase in EEG delta power within the first 2 h, as expected for sleep homeostasis^17,41^, and body temperature returned to basal levels (Extended Data Fig. 2b) (note that, during the first part of the RS period, there was intermittent nesting, and, in those times, body temperature increased, whereas, during individual NREM episodes, body temperature decreased).

**Extended Data Fig. 3:**
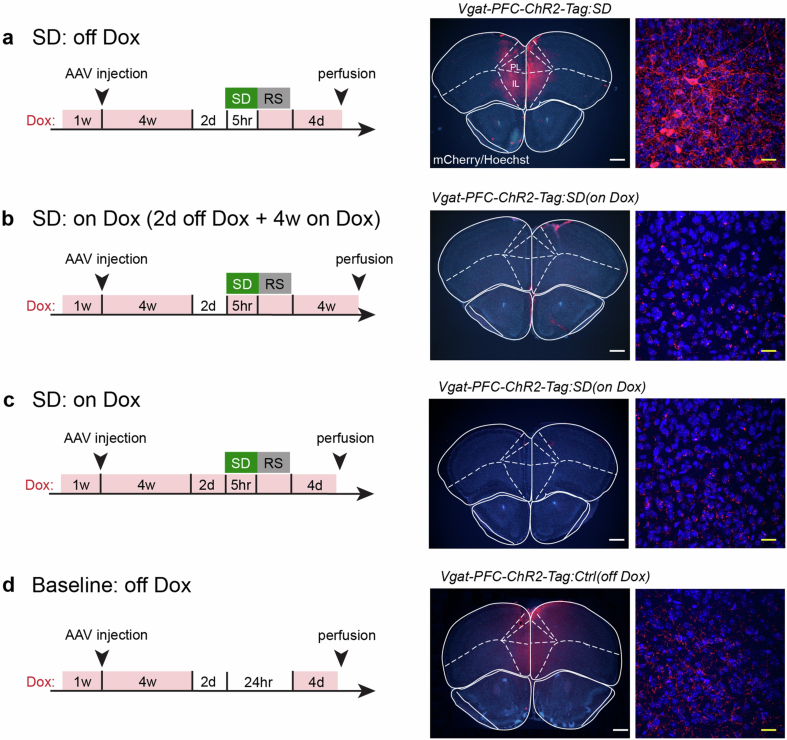
Activity-tagging controls (supports Fig. 2). This Figure shows a series of controls using *Vgat-PFC-hM3Dq-Tag* mice (*n*_*mice*_ = 3 each). All groups of mice were on the Dox diet prior to AAV transgene injection into the PFC. **a**, Experimental group. Mice were on Dox for 4 weeks (4w), then Dox was removed from the diet for 2 days (2d) and during sleep deprivation (SD), and Dox was made available straight after the SD, during the start of nesting and recovery sleep (RS). Four days later, mice were transcardially-perfused and the brain sections were immuno-stained, in this case, for induced mCherry expression in the PFC. Images in the right hand-column are magnifications of PFC cells with induced gene expression**. b**, First type of ‘on Dox’ control group. Mice were on Dox for 4 weeks, then Dox was removed from the diet for 2 days, and then Dox was re-provided during the sleep deprivation procedure and remained in the diet for 4 weeks until transcardio-perfusion. This shows that in the presence of near continuous Dox, except for 2 days interruption, no transgene expression was induced above background. **c**, Second type of ‘on Dox’ control group. Mice were on Dox continuously, including for the periods prior to SD. No transgene expression was induced above background. **d**, Baseline ‘off Dox’ control group. Mice were on Dox for 4 weeks after AAV injection and Dox was removed for 2 days plus an extra 24 hours to correspond with the experimental group, and then mice were placed back on Dox and perfused 4 days later. Although some transgene expression occurred during this time, there was no clear cell labelling at higher magnifications. *n*_*mice*_ = number of biologically independent mice. PL, prelimbic; IL, infralimbic. Scale bars: 100 µm (white), 20 µm (yellow).

Endogenous c-Fos protein was detected in GABAergic cells in both the PFC and the VC after 5 h of SD followed by 2 h of nesting and RS (Extended Data Fig. 2c). Similarly, activity tagging induced human M3 muscarinic (*hM3*)*Dq-mCherry* gene expression in about 32% of PFC^GABA^ cells and 24% of VC^GABA^ cells (Extended Data Fig. 2d). In control *Vgat*-PFC-ChR2-Tag mice, which underwent SD on Dox or experienced baseline activity but not SD off Dox, no transgene expression was present (Extended Data Fig. 3). Thus, c-Fos-driven ChR2 expression was induced in a subset of neocortical GABA cells during SD, nesting and RS in *Vgat*-PFC-ChR2-Tag mice.

### Tagged PFC^GABA^ neurons induce nesting and sleep

**Fig. 2:**
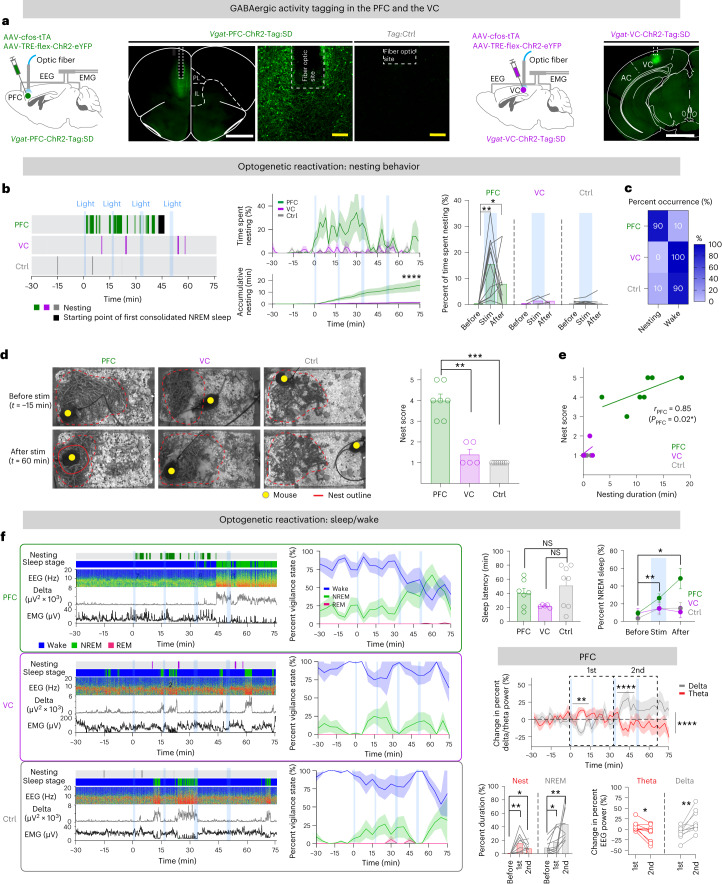
Opto-activation of activity-tagged PFC GABAergic neurons promotes nesting behavior and NREM sleep. **a**, Tagged ChR2–EYFP expression in *Vgat*-PFC-ChR2-Tag:SD, *Vgat*-PFC-ChR2-Tag:Ctrl (Tag:Ctrl) and *Vgat*-VC-ChR2-Tag:SD mice. Cohorts of PFC mice, *n* = 10 sessions, *N* = 7 mice; VC mice, *n* = 6 sessions, *N* = 5 mice; control mice, *n* = 10 sessions; a mix of Tag:Ctrl (*N* = 4) and *Vgat*-PFC-GFP (*N* = 4) mice was used in all panels in this figure, unless otherwise specified. **b**, Nesting activity of optostimulated *Vgat*-PFC-ChR2-Tag:SD (PFC), *Vgat*-VC-ChR2-Tag:SD (VC) and control mice. Left: opto-evoked nesting activity of each cohort. Middle: accumulative time nesting from initiation of optostimulation (ZT 18, *t* = 0 min). Control versus PFC, *P* = 2.10 × 10^−55^; control versus VC, *P* = 4.95 × 10^−1^ with the mixed-effects model. Mean (line) ± s.e.m. (shading). Right: percentage of time in nesting activity before, during and after optostimulation. PFC, before versus during (stim), *P* = 0.002; PFC, before versus after, *P* = 0.0313 with two-tailed Wilcoxon matched-paired signed-rank test. Mean (bars) and before–after individual plot (lines). Blue shading, optostimulation. **c**, Occurrence of consolidated nesting activity during optostimulation. **d**, Nest images before and after optostimulation in all cohorts and nest scores. Yellow dot, mouse position; dashed red line, nesting materials; solid red line, nest. PFC (*n* = 7 sessions) versus control (*n* = 8 sessions), *P* = 0.0002; PFC versus VC (*n* = 5 sessions), *P* = 0.0025 with two-sided Mann–Whitney *U*-test. **e**, Correlation of nest scores and time in nesting activity (two-sided Spearman correlation coefficient). **f**, Left: EEG–EMG traces, nesting activity and sleep stage state, time course of vigilance states. Mouse cohorts are color coded as in **b**. Top right: sleep latency and percentage of NREM sleep before and during optostimulation. PFC versus control, *P* = 0.3204; PFC versus VC, *P* = 0.1616 with two-sided Mann–Whitney *U*-test for sleep latency and PFC before versus stim, *P* = 0.0039; PFC, before versus after, *P* = 0.027 with two-tailed Wilcoxon matched-paired signed-rank test for percent NREM sleep. Middle right: time course of changes in percent delta and theta EEG power against the respective mean baseline (*t* = minus 60 min to 0 min) of *Vgat*-PFC-ChR2-Tag:SD mice before and during optostimulation. Delta versus theta, *P* = 1.50 × 10^−3^ (*t* = 0–16 min), *P* = 6.77 × 10^−5^ (*t* = 32–48 min), *P* = 2.10 × 10^−5^ (*t* = 30–75 min) with two-way RM ANOVA with Bonferroni correction. Bottom right: percent duration and change in percent EEG power of nesting and NREM sleep and theta and delta power of *Vgat*-PFC-ChR2-Tag:SD mice before and during optostimulation. Percent duration (before versus first block, *P* = 0.002 (nest), *P* = 0.0488 (NREM); before versus second block, *P* = 0.0156 (nest), *P* = 0.002), percent power (*P* = 0.0137 (theta), *P* = 0.0039 (delta)) with two-tailed Wilcoxon matched-paired signed-rank test. ‘*t* = 0’, start of optostimulation (blue shading); *n*, number of independent four-bout optostimulation sessions; NS, not significant, *P* ≥ 0.05; *P < 0.05; **P < 0.01; ***P < 0.001; ****P < 0.0001. Mean (line) ± s.e.m. (shading) in time courses, individual plots (circle), mean (bar or circle) ± s.e.m. (error bar) in bar graphs. See also Extended Data Figs. 1–4. PL, prelimbic cortex; IL, infralimbic cortex; AC, auditory cortex. Scale bars, 1,000 µm (**a**, white), 100 µm (**a**, yellow).

**Extended Data Fig. 4:**
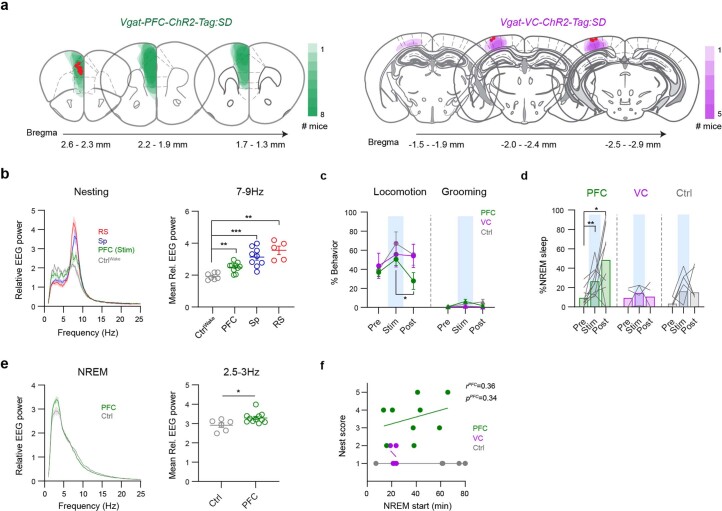
Opto-stimulation of activity-tagged GABA neurons in the PFC and VC (supports Figs. 1, 2). **a**, Map of *ChR2-EYFP* gene expression induced after tagging in *Vgat-PFC-ChR2-Tag:SD* (*N*_*mice*_ = 8) and *Vgat-VC-ChR2-Tag:SD* mice (*N*_*mice*_ = 5). The intensity of the green and purple indicates the extent of overlap (overlay) of gene expression, respectively. Red dot marks the position of the optic fiber tract. **b**, EEG power spectrum of *Vgat-PFC-ChR2-Tag:SD* mice during opto-evoked nesting compared with the EEG spectrum of mice carrying out nesting during the post-SD nesting (RS, red), spontaneous nesting (Sp, green) and Wake EEG spectrum of time-matched *Vgat-PFC-ChR2-Tag:Ctrl* (Ctrl^Wake^, grey). *Right*: Mean EEG power at 7-9 Hz. Ctrl^Wake^ vs. PFC, *P* = 0.0031; Ctrl^Wake^ vs. Sp, *P* = 0.0002; Ctrl^Wake^ vs. RS, *P* = 0.0025 with Two-sided Mann-Whitney *U* test. *Vgat-PFC-ChR2-Tag:SD* (PFC) mice, *N*_*mice*_ = 7, *n*_*session*_ = 10; Ctrl mice (a group of *Vgat-PFC-ChR2-Tag:Ctrl* mice, *N*_*mice*_ = 4, Vgat-PFC-GFP mice, *N*_*mice*_ = 4), *n*_*session*_ = 10;*Vgat-Cre* mice, *N*_*mice*_ = 3, *n*_*session*_ = 9 (Sp), *n*_*session*_ = 5 (RS). **c**, Locomotion and grooming occurrences before and during opto-stimulation for 3 mouse groups (PFC (*n*_*session*_ = 10, *N*_*mice*_ = 7), VC (*n*_*session*_ = 6, *N*_*mice*_ = 5) and control (*n*_*session*_ = 10, *N*_*mice*_ = 8)). *P* = 0.1934 (PFC: Pre vs Stim), *P* = 0.4316 (PFC: Pre vs Post) and *P* = 0.0488 (PFC: Stim vs Post) with Two-tailed Wilcoxon matched-pairs signed rank test for % locomotion. **d**, percentage NREM sleep of *Vgat-PFC-ChR2-Tag:SD* and *Vgat-VC-ChR2-Tag:SD* and control mice before and during opto-stimulation. *P* = 0.0039 (PFC: Pre vs Stim), *P* = 0.1309 (PFC: Stim vs Post) and *P* = 0.0273 (PFC: Pre vs post) with two-tailed Wilcoxon matched-pairs signed rank test. **e**, EEG power spectrum of *Vgat-PFC-ChR2-Tag:SD* and *Vgat-PFC-ChR2-Tag:Ctrl* mice before and during opto-evoked NREM sleep, and mean EEG power at 2.5-3 Hz. *P* = 2.73 × 10^−2^ (PFC vs. Ctrl) with Two-sided Mann-Whitney *U* test. **f**, Correlation of start of NREM sleep and nest score for all 3 groups of mice (Two-sided Spearman correlation coefficient). Mean (line) ± SEM (shading) in **b**, **e**. Individual (open circle), Mean (line/closed circle) ± SEM (error bar) in **b**, **c**, **e**. Mean (bar) and before-after (line) in **d**. *n*_*session*_ = number of independent session, *N*_*mice*_ = number of biologically independent mice. *P < 0.05, **P < 0.01, *** *P* < 0.001.

To examine the roles of these activity-tagged PFC^GABA^ cells, parallel cohorts of *Vgat*-PFC-ChR2-Tag and *Vgat*-VC-ChR2-Tag mice were deprived of sleep and allowed nesting and RS (Fig. 2a and Extended Data Fig. 4a). We termed these mice *Vgat*-PFC-ChR2-Tag:SD and *Vgat*-VC-ChR2-Tag:SD (with the ‘SD’ in these mouse names standing for the collective ‘SD, subsequent nesting and RS’ activities; the control groups of these mice, which had not undergone the tagging behavioral protocol, are labeled ‘Ctrl’). Two days after tagging, optostimulation was directed into the PFC or the VC at ZT 18 (active wake time of mice, ‘lights-off’ period), and behavior and sleep–wake states were recorded (Extended Data Fig. 1b). To ensure stimulating a range of GABAergic neurons that could be involved, we gave a mixed stimulation protocol: 1 min of 10-Hz, 1 min of 20-Hz and two sets of 2 min of 20-Hz light pulses with 15-min intervals, amounting to approximately 50 min of intermittent optostimulation (Extended Data Fig. 1b).

A notable feature was that, during stimulation, *Vgat*-PFC-ChR2-Tag:SD mice interacted with their nesting material much more than *Vgat*-VC-ChR2-Tag:SD mice and the groups of control mice (*Vgat*-PFC-ChR2-Tag:Ctrl and *Vgat*-PFC-YFP mice). For *Vgat*-PFC-ChR2-Tag:SD mice but not *Vgat*-VC-ChR2-Tag:SD or control mice, the time spent nesting during the optostimulation period increased along with an accumulation of built nests (Fig. 2b), with 90% of all optostimulation sessions with *Vgat*-PFC-ChR2-Tag:SD mice showing persistent nesting occurrences during optostimulation (Fig. 2c). Nests built by *Vgat*-PFC-ChR2-Tag:SD mice were of higher quality than those built by other groups (Fig. 2d). Optostimulation-induced nesting durations and nest scores were positively correlated in *Vgat*-PFC-ChR2-Tag:SD mice (*r* = 0.85) (Fig. 2e). During induced nesting in *Vgat*-PFC-ChR2-Tag:SD mice, the EEG power peaked at a theta frequency of 7–9 Hz, similar to that observed for spontaneous nesting^7^ and RS-associated nesting (Extended Data Fig. 4b). On the other hand, during the optostimulation, no other behaviors (locomotion, feeding and grooming) changed (Extended Data Fig. 4c).

Optostimulated *Vgat*-PFC-ChR2-Tag:SD mice entered NREM sleep above baseline levels during the second half of the optostimulation period, coinciding with the time when nesting behavior had decreased, whereas the other groups of mice did not (Fig. 2f and Extended Data Fig. 4d). After 45 min of optostimulation, although the mean sleep latency did not change between *Vgat*-PFC-ChR2-Tag:SD, *Vgat*-VC-ChR2-Tag:SD and control mice (Fig. 2f), the amount of NREM sleep of *Vgat*-PFC-ChR2-Tag:SD mice increased substantially compared with that of other groups (Fig. 2f and Extended Data Fig. 4d). For the first consolidated bout of this NREM sleep, the EEG delta power for optostimulated *Vgat*-PFC-ChR2-Tag:SD mice increased, consistent with this sleep being recapitulated RS (Extended Data Figs. 2b and 4e)^17,41^. The start of NREM sleep in *Vgat*-PFC-ChR2-Tag:SD mice, however, did not correlate with the final quality of the nest (Extended Data Fig. 4f).

**Fig. 3:**
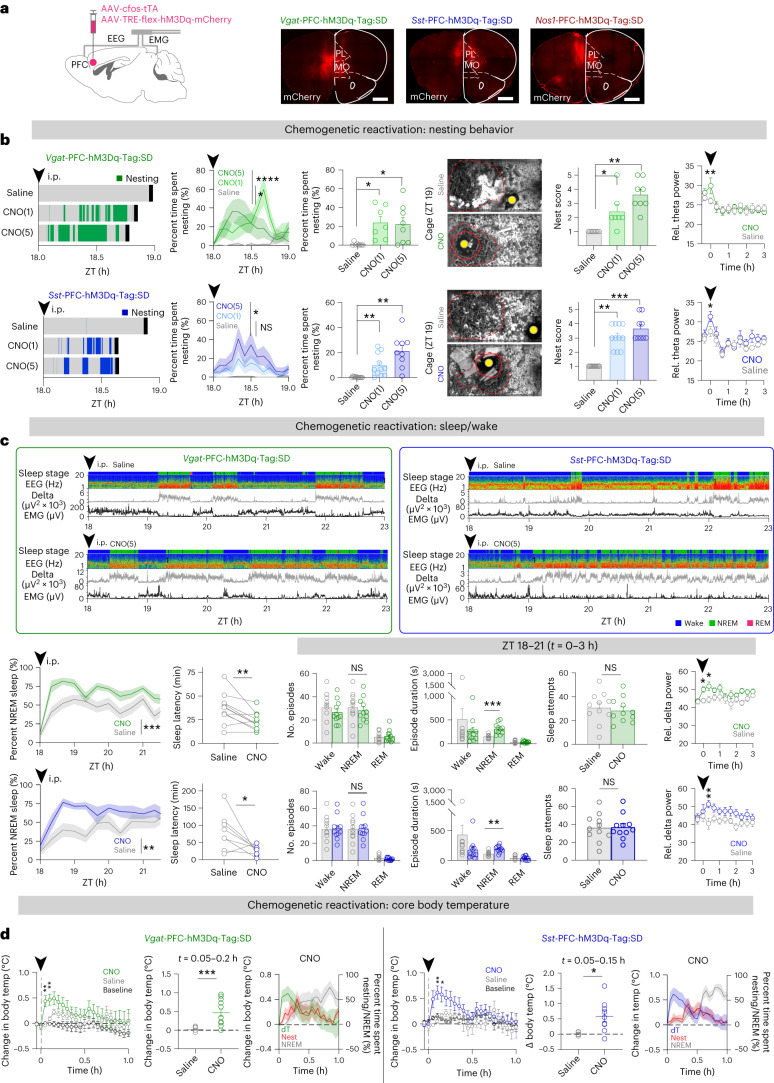
Pharmacogenetic reactivation of tagged GABAergic and *Sst*-expressing neurons in the PFC induces nesting and NREM sleep. **a**, Activity*-*tagged *Vgat*-, *Sst*- and *Nos1*-expressing PFC neurons during SD, nesting activity and RS. Cohorts of *Vgat*-PFC-hM3Dq-Tag:SD mice, *n* = 8 paired sessions, *N* = 8 mice; *Sst*-PFC-hM3Dq-Tag:SD mice, *n* = 12 paired sessions, *N* = 12 mice; *Nos1*-PFC-hM3Dq-Tag:SD mice, *n* = 8 paired sessions, *N* = 8 mice in all panels in this figure, unless specified. **b**, Opto-evoked nesting activity of *Vgat*-PFC-hM3Dq-Tag:SD and *Sst*-PFC-hM3Dq-Tag:SD mice (same mice as in **a**) during the first hour after i.p. injection until the first consolidated sleep (solid black line). i.p. injection of saline and CNO (5 mg per kg, CNO(5); 1 mg per kg, CNO(1)) was at ZT 18 (*t* = 0, solid arrowhead). Other panels, from left to right, time course of percent time spent nesting (saline versus CNO(5), *P* = 0.0394 (*Vgat*), *P* = 0.0178 (*Sst*); saline versus CNO(1), *P* = 2.52 × 10^−5^ (*Vgat*), *P* = 0.262 (*Sst*) with two-way RM ANOVA and Bonferroni correction), nesting duration (saline versus CNO(5), *P* = 0.0156 (*Vgat*), *P* = 0.0039 (*Sst*); saline versus CNO(1), *P* = 0.0156 (*Vgat*), *P* = 0.002 (*Sst*) with two-tailed Wilcoxon matched-paired signed-rank test), representative nest images at ZT 19 (1 h after i.p. injection) (yellow dot, mouse in the cage; dashed red line, nest material outline; solid red line, nest), quantification of nest scores (saline versus CNO(5), *P* = 0.0078 (*Vgat*), *P* = 0.0039 (*Sst*); saline versus CNO(1), *P* = 0.0313 (*Vgat*), *P* = 0.001 (*Sst*) with two-tailed Wilcoxon matched-paired signed-rank test), time course of relative (rel.) theta EEG power (saline versus CNO(5) and CNO(1), *P* = 0.0039 (*Vgat*), *P* = 0.0156 (*Sst*) with two-tailed Wilcoxon matched-paired signed-rank test). **c**, EEG–EMG traces and sleep stage state of *Vgat*-PFC-hM3Dq-Tag:SD and *Sst*-PFC-hM3Dq-Tag:SD mice after saline or CNO(5) i.p. injection. Other panels, from left to right (*P* values with two-tailed Wilcoxon matched-paired signed-rank test unless otherwise specified): NREM sleep time course (*P* = 2.48 × 10^−4^ (*Vgat*), *P* = 3.03 × 10^−3^ (*Sst*) with two-way RM ANOVA and Bonferroni correction), NREM sleep latency (*P* = 0.0068 (*Vgat*), *P* = 0.0391 (*Sst*)), total episode number in ZT 18–21 (*t* = 0–3 h) (*P* = 0.6514 (*Vgat*), *P* > 0.9999 (*Sst*)), mean episode duration (*P* = 0.001 (*Vgat*), *P* = 0.0068 (*Sst*)), sleep attempts (*P* = 0.6514 (*Vgat*), *P* > 0.9999 (*Sst*)) and time course of relative delta NREM EEG power at *t* = 0–3 h (*P* = 0.0156 (*Vgat*, *t* = 0 h), 0.0313 (*Vgat*, *t* = 0.33 h), *P* = 0.0078 (*Sst*, *t* = 0.33 h)). **d**, Core body temperature (temp) change from the pre*-*i.p. time point of *Vgat*-PFC-hM3Dq-Tag:SD (*n* = 6 paired sessions, *N* = 3 mice) and *Sst*-PFC-hM3Dq-Tag:SD (*n* = 6 paired sessions, *N* = 3 mice) mice after i.p. injection (*P* = 0.007 (*Vgat*, *t* = 0.05 h), *P* = 0.0066 (*Vgat*, *t* = 0.1 h), *P* = 0.0082 (*Sst*, *t* = 0.1 h), *P* = 0.0398 (*Sst*, *t* = 0.15 h) with two-tailed Wilcoxon matched-paired signed-rank test) and mean change in body temperature at *t* = 0.05–0.20 h in *Vgat*-PFC-hM3Dq-Tag:SD (*P* = 0.0008) and *t* = 0.05–0.15 h in *Sst*-PFC-hM3Dq-Tag:SD (*P* = 0.019) mice with two-sided Mann–Whitney *U*-test*.* *n*, number of paired i.p. experiment sessions. NS, not significant, *P* ≥ 0.05; *P < 0.05; **P < 0.01; ***P < 0.001; ****P < 0.0001. Mean (line) ± s.e.m. (shading) in **b**–**d**. Individual points (open circles), mean (bar) and s.e.m. (error bar) in bar graphs in **b**–**d**. See also Extended Data Figs. 1 and 5. MO, medial orbital cortex. Scale bar, 1,000 µm (**a**).

**Extended Data Fig. 5:**
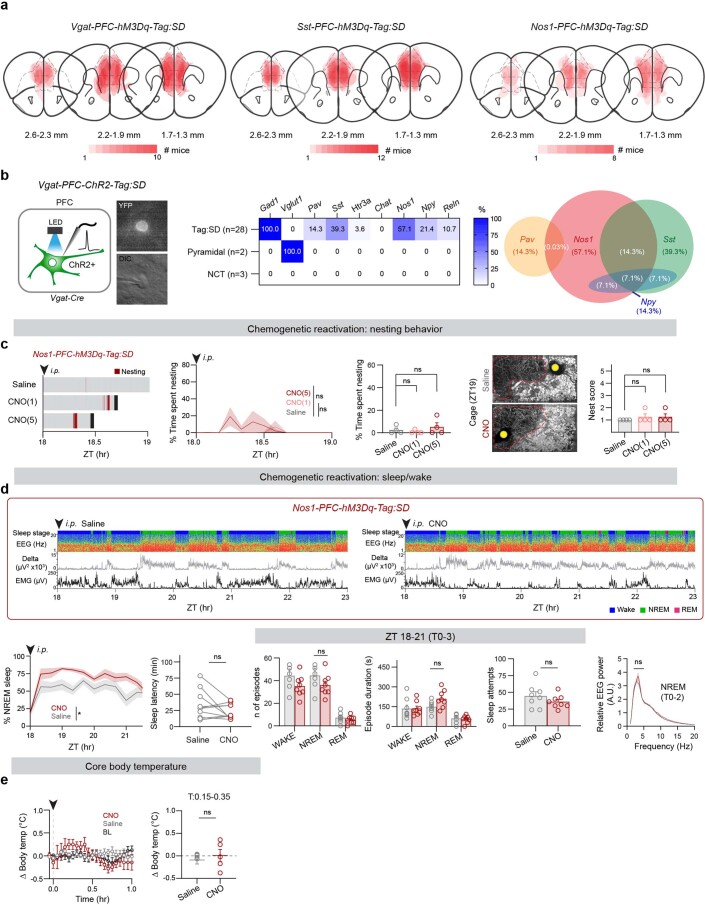
Activity-tagging and chemogenetic stimulation of PFC^*Vgat*^, PFC^*Sst*^, and PFC^*Nos1*^ neurons, genetic characterization of the tagged Vgat neurons, and further data on sleep characterization (Supports Fig. 3). **a**, Schematic map of *hM3Dq-mCherry* gene expression induced after tagging in *Vgat-PFC-hM3Dq-Tag:SD* (*n*_*mice*_ = 10)*, Sst-PFC-hM3Dq-Tag:SD* (*n*_*mice*_ = 12) *and Nos1-PFC-hM3Dq-Tag:SD* (*n*_*mice*_ = 8) in the PFC (intensity of red, indicates extent of overlap between animals). **b**, Single-cell RT-PCR from visually-identified tagged neurons in acute PFC slices prepared from *Vgat-PFC-ChR2-Tag:SD* mice (*N*_*mice*_ = 6). Dark blue, strongest expression; pale blue, weaker. NCT, no template control. Venn diagram showing that many *Gad1*-expressing PFC neurons in *Vgat-PFC-ChR2-Tag:SD* mice co-express *Sst* and/or *Nos1*. *N*_*neuron*_ = 28 (ChR2^+^). **c**, Evoked nesting activity of *Nos1-PFC-hM3Dq-Tag:SD* mice that had received SD and then saline/CNO injections 2 days later. Red raster bars, nesting during first 1 hour of post-*i.p*. injection until the 1^st^ consolidated sleep occurrence (solid black line); *i.p*. injection was at ZT18 (solid arrowhead). The other panels from left to right: time course of %time spent nesting (Group x time, *P* = 1.40 × 10^−1^ (saline vs. CNO (5)), *P* > 0.9999 (saline vs. CNO(1)) with 2-way RM ANOVA with Bonferroni correction), nesting duration (*P* = 0.75 (saline vs. CNO (5)), *P* = 0.375 (saline vs. CNO(1)) with two-tailed Wilcoxon matched-paired signed rank test), pictures of scattered nesting material and nests (Yellow dot, mouse in the cage; dashed red line, nest material outline; solid red line, nest outline), quantification of nest scores (*P* > 0.9999 (saline vs. CNO(5)), *P* > 0.9999 (saline vs. CNO(1))) with two-tailed Wilcoxon matched-paired signed rank test, *n*_*paired-sessions*_ = 4, *N*_*mice*_ = 4. **d**, *Top:* Example EEG/EMG trace and sleep stage state of *Nos1-PFC-hM3Dq-Tag:SD* after saline and CNO *i.p*. injection. *Bottom*: from left to right, NREM sleep time course (*P* = 4.62 × 10^−2^ with 2-way RM ANOVA with Bonferroni correction), NREM sleep latency (*P* = 0.4922), total number of episodes in first 3 hours post-*i.p*. injection (T:0-3, NREM: *P* = 0.1563), mean episode duration per hour (NREM: *P* = 0.0547), sleep attempts (*P* = 0.156) with two-tailed Wilcoxon matched-paired signed rank test and NREM EEG spectrum of T:0-2 (*P* = 6.11 × 10^−1^) with 2-way RM ANOVA with Bonferroni correction. *n*_*paired-session*_ = 10, *N*_*mice*_ = 8. Mean (line) ± SEM (shading), Individual points (open circle), Mean (bar) ± SEM (error bar). **e**, core body temperature change from pre*-i.p*. time point of *Nos1-PFC-hM3Dq-Tag:SD* after saline and CNO *i.p*. injection and mean change in body temperature at T:0.05-0.35 period. *P* = 0.7922 with two-sided Mann-Whitney *U* test. *n*_*paired-session*_ = 6, *N*_*mice*_ = 3. ^ns^*P* ≥ 0.05, *P < 0.05. Individual points (open circle), Mean (line) ± SEM (error bar). *n*_*paired-session*_ = number of independent paired-sessions, *N*_*mice*_ = number of biologically independent mice.

We repeated the tagging experiments using chemogenetics with *Vgat*-PFC-hM3Dq-Tag mice (Fig. 3a and Extended Data Figs. 1c and 5a). Two days after tagging, an intraperitoneal (i.p.) injection of clozapine-*N*-oxide (CNO) (1 or 5 mg per kg) at ZT 18 elicited prolonged nesting, high nest-quality scores and increased theta power in EEG (Fig. 3b); sustained NREM sleep was induced within 1 h compared with saline-injected activity-tagged mice (Fig. 3c). Thus, the chemogenetic and optogenetic results were in the same direction.

Summarizing thus far, of the four consecutive blocks of optostimulation sessions, the first and second sessions in *Vgat*-PFC-ChR2-Tag:SD mice induced nesting behaviors, whereas the third and four sessions induced NREM sleep (Fig. 2f). Consistently, theta power in EEG was elevated in the first two blocks of optostimulation, whereas delta power increased in the second two blocks of optostimulation (Fig. 2f). This suggests that the reactivated tagged neurons or circuitry successfully recapitulate the behaviors (nesting and sleep) in the tagging procedure (Fig. 1a,b).

### Tagged PFC^*Sst*^ cells induce nesting and sleep

As assessed by c-Fos immunohistochemistry, *Nos1*-expressing cells become active throughout the neocortex during RS^25–27^. Neocortical *Nos1*-expressing cells are a subset of *Sst*-expressing GABA neurons^42^. Tagged PFC neurons were analyzed by single-cell multiplex real-time (RT)–qPCR in acute slices of the PFC from *Vgat*-PFC-ChR2-Tag:SD mice (ChR2^+^, 28 cells, six animals) (Extended Data Fig. 5b). Tagged cells all expressed *Gad1* (encoding glutamic acid decarboxylase 1; Extended Data Fig. 5b). Of these tagged cells, there was a mixture of *Sst*- and *Nos1*-positive cells (*Nos1* only, *Sst* only or both genes coexpressed, Extended Data Fig. 5b). Pyramidal cells in *Vgat*-PFC-ChR2-Tag:SD mice were green fluorescent protein (GFP)-negative and expressed *Vglut1* (*Slc17a7*) but not *Gad1* (Extended Data Fig. 5b).

To explore the function of *Sst-* and *Nos1*-expressing cells responding to activity tagging, we generated *Sst*-PFC-hM3Dq-Tag and *Nos1*-PFC-hM3Dq-Tag mice; *Vgat*-PFC-hM3Dq-Tag mice served as a comparison (Fig. 3a and Extended Data Fig. 5a). In both *Sst*-PFC-hM3Dq-Tag:SD and *Nos1*-PFC-hM3Dq-Tag:SD mice, there was an induction of c-Fos*-*dependent hM3Dq–mCherry expression in the PFC (Fig. 3a). As for the *Vgat*-PFC-hM3Dq-Tag:SD mice described above, 2 d after tagging, an i.p. injection of CNO (1 or 5 mg per kg) at ZT 18 elicited prolonged nesting behavior and high nest-quality scores and enhanced theta power in *Sst*-PFC-hM3Dq-Tag:SD mice (Fig. 3b); however, there was no increase in nesting behavior for *Nos1*-PFC-hM3Dq-Tag:SD mice (Extended Data Fig. 5c).

For *Sst*-PFC-hM3Dq-Tag:SD mice, after CNO injection and the nesting activity described above, the latency to NREM sleep was reduced, as found for *Vgat*-PFC-hM3Dq-Tag:SD mice (Fig. 3c), whereas, for *Nos1*-PFC-hM3Dq-Tag:SD mice, NREM sleep latency was unchanged (Extended Data Fig. 5d). For *Vgat*-PFC-hM3Dq-Tag:SD and *Sst*-PFC-hM3Dq-Tag:SD mice, sustained NREM sleep was induced above baseline compared with saline in *Sst*-PFC-hM3Dq-Tag:SD mice, as for the *Vgat*-PFC-hM3Dq-Tag:SD mice (Fig. 3c); for both sets of mice, the number of sleep attempts and episodes did not change, but only the duration of NREM episodes was prolonged (Fig. 3c). Delta power of evoked NREM sleep was increased in the first hour (Fig. 3c), consistent with this sleep being RS^41^. By contrast, for *Nos1*-PFC-hM3Dq-Tag:SD mice, NREM induction, although statistically significant, was not sustained (Extended Data Fig. 5d), and there were no changes in sleep latency, sleep attempts, numbers of episodes or episode duration of NREM sleep (Extended Data Fig. 5d). For both *Vgat*-PFC-hM3Dq-Tag:SD and *Sst*-PFC-hM3Dq-Tag:SD mice, the core body temperature increased within 5 min of CNO i.p. injection compared with mice treated with saline, before nesting (Fig. 3d), but, when NREM sleep started some 30 min later, temperature decreased (Fig. 3d). For *Nos1*-PFC-hM3Dq-Tag:SD mice injected with CNO, however, there were no temperature changes (Extended Data Fig. 5e). Therefore, of the types of *Vgat*-expressing cell types studied after SD, only the PFC^*Sst*^ cells induced nesting, temperature changes and RS.

**Fig. 4:**
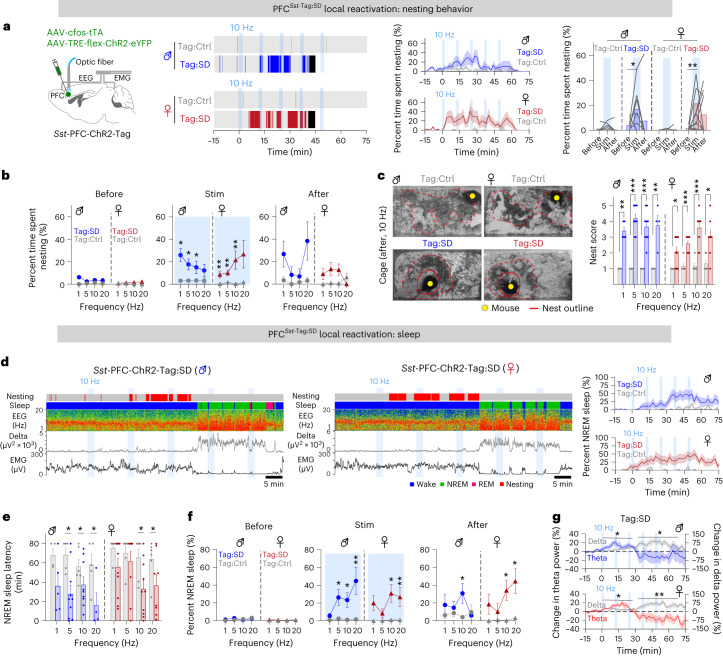
Reactivation of tagged PFC^*Sst*^ neurons induces nesting and NREM sleep in both female and male mice. **a**, *Sst*-PFC-ChR2-Tag:SD mice and optostimulation of the PFC soma and nesting activity of optostimulated *Sst*-PFC-ChR2-Tag:SD (Tag:SD) and *Sst*-PFC-ChR2-Tag:Ctrl (Tag:Ctrl, paired on-Dox control) male and female mice during optostimulation at 10 Hz. Animal cohorts of *n* = 8 sessions and *N* = 4 mice for each sex. Left: opto-evoked nesting activity. Start of first consolidated NREM sleep is shown with a black line. Right: percentage time spent nesting over time and before, during and after optostimulation at 10 Hz from *t* = 0, the starting point of optostimulation (ZT 18). Before versus stim (*P* = 0.0313 (Tag:SD, male), *P* = 0.0098 (Tag:SD, female) with two-tailed Wilcoxon matched-paired signed-rank test). **b**, How optostimulation frequencies in the PFC elicit time spent nesting for *Sst*-PFC-ChR2-Tag:SD mice and their paired on-Dox controls. Stim (Tag:Ctrl versus Tag:SD), male: *P* = 0.0159 (1 Hz), *P* = 0.0121 (5 Hz), *P* = 0.0316 (10 Hz), *P* = 0.246 (20 Hz); female: *P* = 0.0035 (1 Hz), *P* = 0.0067 (5 Hz), *P* = 0.0044 (10 Hz), *P* = 0.1177 (20 Hz) with two-sided Mann–Whitney *U*-test. **c**, Nests after five bouts of 10-Hz stimuli for *Sst*-PFC-ChR2-Tag:SD mice and paired on-Dox controls. Tag:Ctrl versus Tag:SD, male: *P* = 0.0079 (1 Hz), *P* = 0.0003 (5 Hz), *P* = 0.0002 (10 Hz), *P* = 0.0079 (20 Hz); female: *P* = 0.0264 (1 Hz), *P* = 0.0007 (5 Hz), *P* = 0.0004 (10 Hz), *P* = 0.0117 (20 Hz) with two-sided Mann–Whitney *U*-test. **d**, EEG–EMG traces, sleep stage state and time course of percentage NREM sleep before and after 10-Hz optostimulation in the PFC for male and female *Sst*-PFC-ChR2-Tag:SD mice. **e**, NREM sleep latency of *Sst*-PFC-ChR2-Tag:SD mice and paired on-Dox controls with various optostimulation frequencies. Tag:Ctrl versus Tag:SD, male: *P* = 0.1032 (1 Hz), *P* = 0.0137 (5 Hz), *P* = 0.036 (10 Hz), *P* = 0.0317 (20 Hz); female: *P* = 0.9176 (5 Hz), *P* = 0.0287 (10 Hz), *P* = 0.0303 (20 Hz) with two-sided Mann–Whitney *U*-test. **f**, Effect of different optostimulation frequencies in the PFC in eliciting percent time spent in NREM sleep for *Sst*-PFC-ChR2-Tag:SD mice and their paired on-Dox controls. Tag:Ctrl versus Tag:SD, male: *P* = 0.041 (stim, 5 Hz), *P* = 0.0216 (stim, 10 Hz), *P* = 0.0083 (stim, 20 Hz), *P* = 0.0216 (after, 10 Hz); female: *P* = 0.1285 (stim, 1 Hz), *P* = 0.0387 (stim, 10 Hz), *P* = 0.0022 (stim, 20 Hz), *P* = 0.0374 (after, 10 Hz), *P* = 0.0281 (after, 20 Hz) with two-sided Mann–Whitney *U*-test. **g**, Change in theta and delta EEG power from baseline during 10-Hz optostimulation of *Sst*-PFC-ChR2-Tag:SD mice. Tag:Ctrl versus Tag:SD, male: *P* = 0.0147 (delta, *t* = 30–70 min), *P* = 0.0394 (theta, *t* = 0–30 min); female: *P* = 0.0098 (delta, *t* = 30–70 min), *P* = 0.049 (theta, *t* = 0–30 min) with the mixed-effects model. *n*, number of independent five-bout optostimulation sessions. *P < 0.05; **P < 0.01; ***P < 0.001. Mean (line) ± s.e.m. (shading) in **a**,**d**,**g**. Individual plots (before–after, line) and mean (bar) in **a**. Mean (circle or triangle) ± s.e.m. (line) in **b**,**f**. Individual plot (dot), mean (bar) and +s.e.m. (error bar) in **c**,**e**. See also Extended Data Figs. 1 and 6.

**Extended Data Fig. 6:**
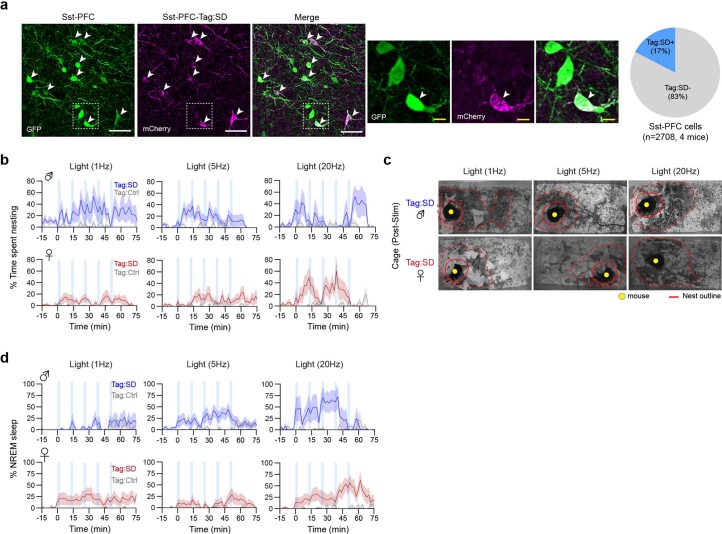
PFC^*Sst*^ optogenetics (Supports Fig. 4). **a**, Percentage of tagged PFC^*Sst*^ neurons in the PFC as assessed by *hM3Dq-mCherry* expression (mCherry in magenta). PFC^*Sst*^ neurons are defined by *ChR2-EYFP* expression after co-injecting *AAV-flex-ChR2-EYFP* into the PFC of *Sst-Cre* mice. Arrowheads indicate co-expression. *Sst-PFC-YFP::Sst-PFC-hM3Dq-Tag:SD* mice, *n*_*neuron*_ = 2708, *N*_*mice*_ = 4. **b**, Time spent nesting with 1 Hz, 5 Hz, or 20 Hz light stimulation of PFC for male and females *Sst-PFC-ChR2-Tag:SD* mice and their controls. **c**, Example pictures of nests after 5 bouts of 1, 5, or 20 Hz stimuli for male and female *Sst-PFC-ChR2-Tag:SD* mice. Yellow dot, mouse in the cage; dashed red line, nest material outline; solid red line, nest outline. **d**, time spent in NREM sleep with 1 Hz, 5 Hz, or 20 Hz light stimulation of PFC for male and female *Sst-PFC-ChR2-Tag:SD* mice and their controls. *n*_*neuron*_ = number of neurons, *N*_*mice*_ = number of biologically independent mice. Mean (line) ± SEM (shading). Scale bars; 50 µm (in white), 10 µm (in yellow).

Focusing on the PFC^*Sst*^ subset of GABA cells, we confirmed the chemogenetic results using opto-activation with *Sst*-PFC-ChR2-Tag:SD male and female mice (Fig. 4a). After tagging, 17% of PFC^*Sst*^ cells were labeled with hM3Dq (Extended Data Fig. 6a). Optostimulation was directed into the PFC of *Sst*-PFC-ChR2-Tag:SD animals as a session of five bouts of 2 min at various frequencies (1, 5, 10, 20 Hz) with a 10-min interstimulus interval (Fig. 4a and Extended Data Fig. 1b). The behavioral baseline evoked by optostimulation was obtained on the same animals before the tagging procedure (*Sst*-PFC-ChR2-Tag:Ctrl mice). For all frequencies, for both males and females, the time spent nesting during the optostimulation period was longer in *Sst*-PFC-ChR2-Tag:SD mice than that in *Sst*-PFC-ChR2-Tag:Ctrl mice (Fig. 4a,b and Extended Data Fig. 6b). At the end of the stimulations, nest scores in both sexes were higher than those achieved by control mice for all stimulation frequencies (Fig. 4c and Extended Data Fig. 6c).

As with *Vgat*-PFC-ChR2-Tag:SD mice, optostimulated *Sst*-PFC-ChR2-Tag:SD mice entered NREM sleep above baseline levels during the second half of the optostimulation period, coinciding with the time when nesting behavior had decreased (Fig. 4d and Extended Data Fig. 6d). During optostimulation, the mean NREM sleep latency decreased in *Sst*-PFC-ChR2-Tag:SD mice (Fig. 4e) and the amount of NREM sleep exhibited by *Sst*-PFC-ChR2-Tag:SD mice increased substantially compared with that of *Sst*-PFC-ChR2-Tag:Ctrl mice (Fig. 4f), and this sleep persisted after optostimulation at 10 and 20 Hz (Fig. 4f and Extended Data Fig. 6d). As with *Vgat*-PFC-ChR2-Tag:SD mice, optostimulation in *Sst*-PFC-ChR2-Tag:SD mice first increased theta power, followed by an increase in delta power (Fig. 4g). Therefore, PFC^*Sst*^ cells that are captured by a combination of SD, nesting and RS can initiate nesting and NREM sleep similar to PFC^*Vgat*^ cells, and this opto-induced behavior change does not depend on sex.

### Tagged PFC^*Sst*^ cells fire rapidly and target the hypothalamus

**Fig. 5:**
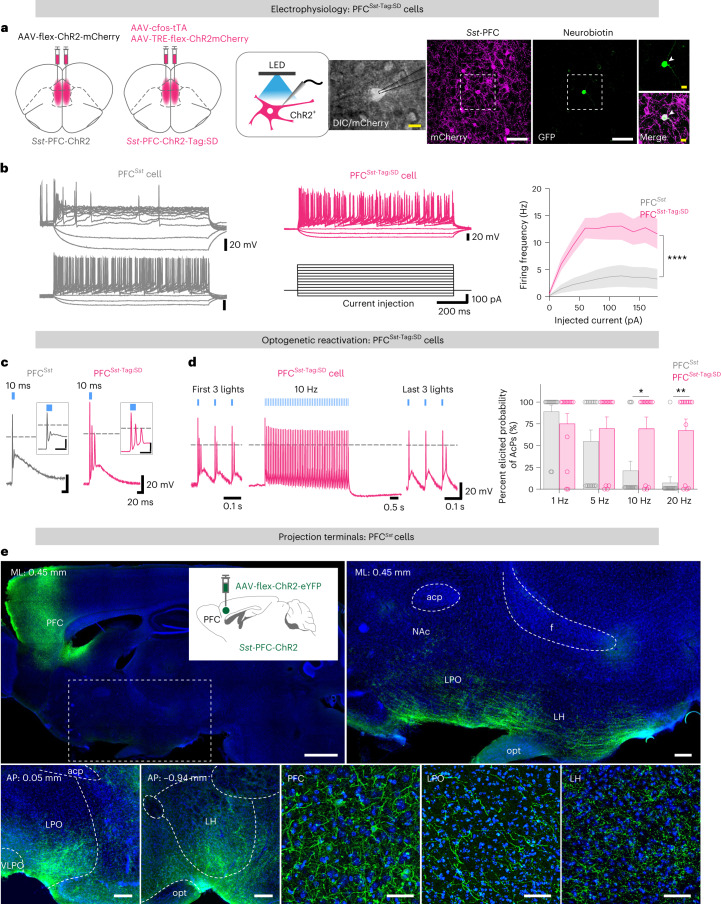
PFC^*Sst*^ cells activated by the tagging protocol are fast spiking and project to the preoptic hypothalamus and the LH. **a**, A patched PFC^*Sst*^ cell from an *Sst*-PFC-ChR2-Tag:SD mouse and electrode patching an mCherry-positive cell. **b**, Current-clamp recordings showing membrane voltage changes of randomly sampled PFC^*Sst*^ cells from *Sst*-PFC-ChR2 mice (PFC^*Sst*^ cells, gray) and tagged PFC^*Sst*^ cells from *Sst*-PFC-ChR2-Tag:SD mice (PFC^*Sst*-Tag:SD^ cells, magenta). Right: action potential (spike) frequency following different current injections. PFC^*Sst*^ versus PFC^*Sst*-Tag:SD^, *P* = 4.00 × 10^−8^ with the mixed-effects model. Cohorts of *Sst*-PFC-ChR2 mice, *n* = 15 neurons, *N* = 6 mice; *Sst*-PFC-ChR2-Tag:SD mice, *n* = 13 neurons, *N* = 7 mice in all panels in this figure. **c**, Action potentials triggered by a 10-ms light stimulus to randomly sampled PFC^*Sst*^ cells (gray) and PFC^*Sst*-Tag:SD^ cells (magenta). **d**, Action potential responses following a 1-s train of 10-ms pulses at 10 Hz to PFC^*Sst*-Tag:SD^ cells (magenta). Right-hand graph: elicited probabilities of light-evoked action potentials (AcPs) according to optostimulation frequency. PFC^*Sst*^ versus PFC^*Sst*-Tag:SD^, *P* = 0.0297 (10 Hz), *P* = 0.0043 (20 Hz), two-sided Mann–Whitney *U*-test. **e**, Sagittal (top) and coronal brain sections (bottom) from *Sst*-PFC-ChR2 mice (*N* = 5 mice) showing axons (labeled green by ChR2–EYFP) extending into the LPO hypothalamus and the LH. *n*, number of neurons*.* *P < 0.05; **P < 0.01; ****P <0.0001. Mean (line) ± s.e.m. (shading) in **b**; individual plot (open circle), mean (bar) + s.e.m. (line) in **d**. See also Extended Data Figs. 7 and 8. Acp, anterior commissure, posterior part; f, fornix; NAc, nucleus accumbens; opt, optic tract; VLPO, ventral LPO hypothalamus. Scale bars, 50 µm (**a**, white), 10 µm (**a**, yellow), 1,000 µm (**e**, sagittal, left), 200 µm (**e**, sagittal, right), 100 µm (**e**, coronal, left), 25 µm (**e**, coronal, right).

**Extended Data Fig. 7:**
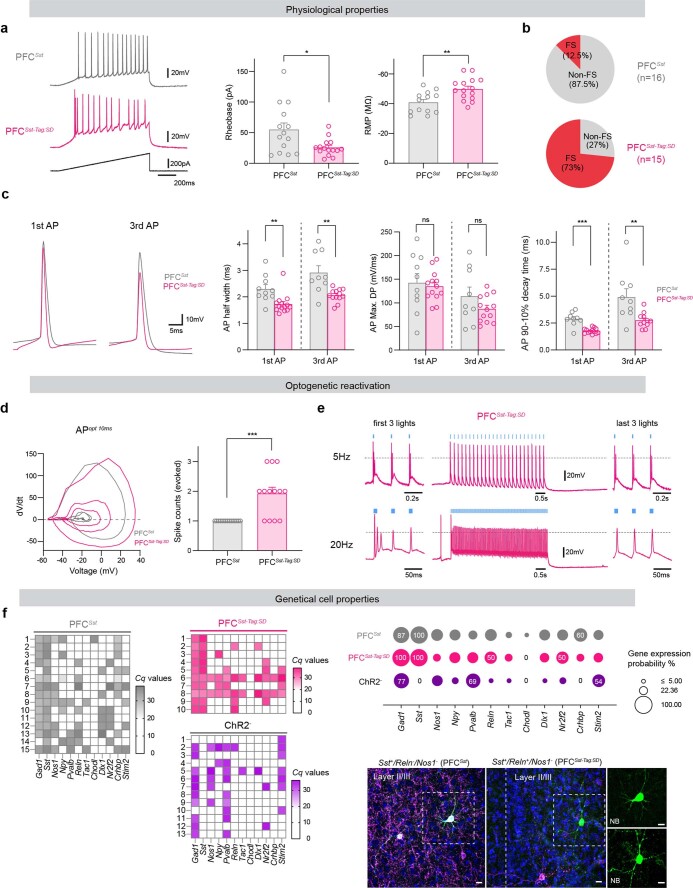
Electrophysiological properties and gene expression phenotypes of tagged and non-tagged PFC^Sst^ cells (Supports Fig. 5). **a**, Current-clamp recording traces in a ramp test and basic cell properties of randomly-sampled PFC^*Sst*^ cells from *Sst-PFC-ChR2* mice (PFC^*Sst*^ cells, grey) and tagged PFC^*Sst*^ cells from *Sst-PFC-ChR2-Tag:SD* mice (PFC^*Sst-Tag:SD*^ cells, magenta). *P* = 0.047 (Rheobase), *P* = 0.0071 (RMP).Cohorts of PFC^*Sst*^: *n*_*neuron*_ = 16, *Sst-PFC-ChR2* mice (*N*_*mice*_ = 6), PFC^*Sst-Tag:SD*^: *n*_*neuron*_ = 15, *Sst-PFC-ChR2-Tag:SD* mice (*N*_*mice*_ = 7). **b**, Proportions of fast-spiking cells (FS) within all sampled cells from both PFC^*Sst*^ cells and PFC^*Sst-Tag:SD*^ cells. **c**, Action potential (AP) properties of 1^st^ and 3^rd^ evoked APs after giving 60 pA steps in PFC^*Sst*^ cells and PFC^*Sst-Tag:SD*^ cells. Left panel: example trace overlaps of PFC^*Sst*^ cells and PFC^*Sst-Tag:SD*^ cells. AP half width: *P* = 0.0015 (1^st^), *P* = 0.0014 (3^rd^), AP Max. DP: *P* = 0.681 (1^st^), *P* = 0.4707 (3^rd^), AP 90-10% decay time: *P* = 0.0006 (1^st^), *P* = 0.0074 (3^rd^). **d**, Phase plot and evoked spike counts of action potentials triggered by a single 10 ms blue light pulse in PFC^*Sst*^ cells and PFC^*Sst-Tag:SD*^ cells. *P* = 0.0001. **e**, example of action potential responses following a 1 sec train of 10 msec pulses of blue light delivered at 5 Hz and 20 Hz to PFC^*Sst-Tag:SD*^ cells. **F**, gene expression matrix of PFC^*Sst*^ cells (in grey), PFC^*Sst-Tag:SD*^ cells (magenta) and ChR2-mCherry^-^ cells from *Sst-PFC-ChR2* mice and *Sst-PFC-ChR2-Tag:SD* mice (purple). Image shows Layer2/3 cells filled post-recording with neurobiotin (NB). Two-sided Mann-Whitney *U* test. ^ns^*P* ≥ 0.05, *P < 0.05, ** *P* < 0.01, *** *P* < 0.001. *n*_*neuron*_ = number of neurons. *N*_*mice*_ = number of biologically independent mice. Individual plot (open circle), Mean (bar) ± SEM (error bar). Scale bars, 10 µm.

We characterized the electrophysiological identities of the tagged PFC^*Sst*-GABA^ neurons in *Sst*-PFC-ChR2-Tag mice. Acute slices of the PFC were prepared 2 d after SD, and ChR2–mCherry^+^ cells were whole-cell patch clamped (Fig. 5a,b). As a control, to sample the diversity of PFC^*Sst*^ cells, AAV-flex-ChR2-mCherry virus was injected into the PFC of *Sst*^Cre^ mice (*Sst*-PFC-ChR2 mice), and *Sst*-ChR2–mCherry^+^ cells were patched randomly. The electrophysiological parameters of the tagged PFC^*Sst*^ cells (PFC^*Sst*-Tag:SD^ cells) differed from those of control PFC^*Sst*^ cells from *Sst*-PFC-ChR2 mice (PFC^*Sst*^ cells): although the resting membrane potentials of tagged cells were more hyperpolarized, their rheobase, the amount of current needed to elicit threshold firing of action potentials, was lower (Extended Data Fig. 7a). There was also a marked difference in the way that the two groups of cells (PFC^*Sst*-Tag:SD^ or PFC^*Sst*^ cells) were able to fire action potentials. A series of current injections were made to test cell excitability (Fig. 5b). About 70% of PFC^*Sst*-Tag:SD^ cells could consistently fire action potentials at higher frequencies (15 Hz; Fig. 5b and Extended Data Fig. 7b), whereas PFC^*Sst*^ cells showed a range of electrophenotypes: most were slow spiking (5 Hz), but a minority (12.5%) were fast spiking (Fig. 5b and Extended Data Fig. 7b), as reported from sampling *Sst*-expressing cells in the mouse VC^42^. Evoked action potentials from PFC^*Sst*-Tag:SD^ cells were narrower (that is, more briefly lasting; the rising time was the same, but action potential half-width and decay time were reduced) than those from randomly sampled *Sst*-expressing cells (Extended Data Fig. 7c). Furthermore, these PFC^*Sst*-Tag:SD^ cells differed in how they responded to optostimulation. When identified cells were given a single 10-ms light pulse, PFC^*Sst*-Tag:SD^ cells produced doublet or triplet action potentials, whereas randomly sampled PFC^*Sst*^ cells did not, only giving single action potentials (Fig. 5c and Extended Data Fig. 7d). When PFC^*Sst*-Tag:SD^ cells were given the range of optostimulation frequencies that elicit nesting and sleep in vivo, the cells sustained multiple spikes at all stimulation frequencies (1, 5, 10, 20 Hz) (Fig. 5d and Extended Data Fig. 7e). Overall, tagging captured particularly excitable, fast-spiking *Sst-*expressing GABA cells, a recently discovered type of *Sst*-expressing neocortical cell^42^.

We next characterized, using single-cell multiplex RT–qPCR, the gene expression profile of these PFC^*Sst*-Tag:SD^ cells versus randomly sampled PFC^*Sst*^ cells (Extended Data Fig. 7f). As expected, for both the PFC^*Sst*-Tag:SD^ and PFC^*Sst*^ cells, the predominant transcripts detected were *Sst* and *Gad1*. Few PFC^*Sst*-Tag:SD^ cells, however, expressed *Nos1*, and none expressed *Chodl* (encoding chondrolectin), whereas these two genes (*Nos1* and *Chodl*) were expressed in randomly sampled PFC^*Sst*^ cells. A third of PFC^*Sst*-Tag:SD^ cells expressed *Pvalb* (encoding parvalbumin), and about half expressed *Reln* (encoding reelin). PFC^*Sst*-Tag:SD^ cells were mainly in layers 2–3 and 5 (Extended Data Fig. 7f).

**Extended Data Fig. 8:**
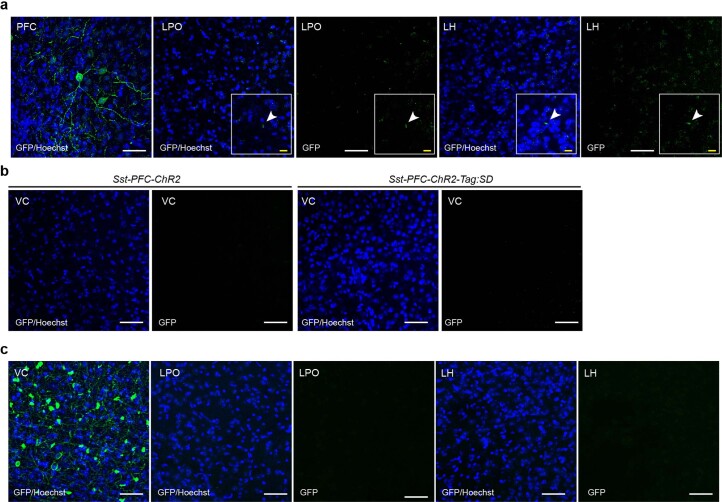
Detection of ChR2-EYFP signals in the LPO and LH of Sst-PFC-ChR2-Tag:SD and Sst-VC-ChR2 mice (Supports Fig. 5). **a**, Images from *Sst-PFC-ChR2-Tag:SD* mice (*N*_*mice*_ = 5); three tagged ChR2-EYFP-positive cell bodies are shown in PFC, and fine processes (green) in LPO and LH, reflecting a transient bolus of ChR2-EYFP. Arrowhead, an axon terminal. **b**, Images from *Sst-PFC-ChR2-Tag:SD* and *Sst-PFC-ChR2* mice showing no ChR2-EYFP labelled fibers in VC (*N*_*mice*_ = 5 each). **c**, Images from *Sst-VC-ChR2* mice (*N*_*mice*_ = 3) showing multiple ChR2-EYFP-positive cell bodies in the VC itself, but no labelled fibers in LPO or the LH. *N*_*mice*_ = number of biologically independent mice. Scale bars; 50 µm (**a**, white), 10 µm (**a**, yellow), 50 µm (**b**, **c**).

We examined the projections of these PFC^*Sst*-Tag:SD^ cells. Although fluorescence was mainly in the PFC, both the LH (ventral part) and the LPO hypothalamus (ventral part) contained fine ChR2–enhanced yellow fluorescent protein (EYFP)^+^ fibers (Extended Data Fig. 8a). We also examined constitutively (non-tagged) labeled cells from *Sst*-PFC-ChR2 mice (Fig. 5e) and found long-range projections of labeled axons from PFC^*Sst*^ neurons. These axons did not cross the midline. Many fibers could be seen in the LPO hypothalamus and the LH (Fig. 5e) but not in areas of the cortex beyond the PFC, similar to *Sst*-PFC-Tag:SD signals (Extended Data Fig. 8b). There were no fibers, however, in the base of the brain from labeled VC^*Sst*^ cells constitutively expressing ChR2–EYFP in *Sst*-VC-ChR2 mice (Extended Data Fig. 8c). We focused on the preoptic hypothalamus and the LH as areas potentially involved in sleep and/or nesting behaviors mediated by PFC^*Sst*-GABA^ cells.

### PFC^*Sst*-GABA^ terminals in the LPO hypothalamus induce nesting but not sleep

**Fig. 6:**
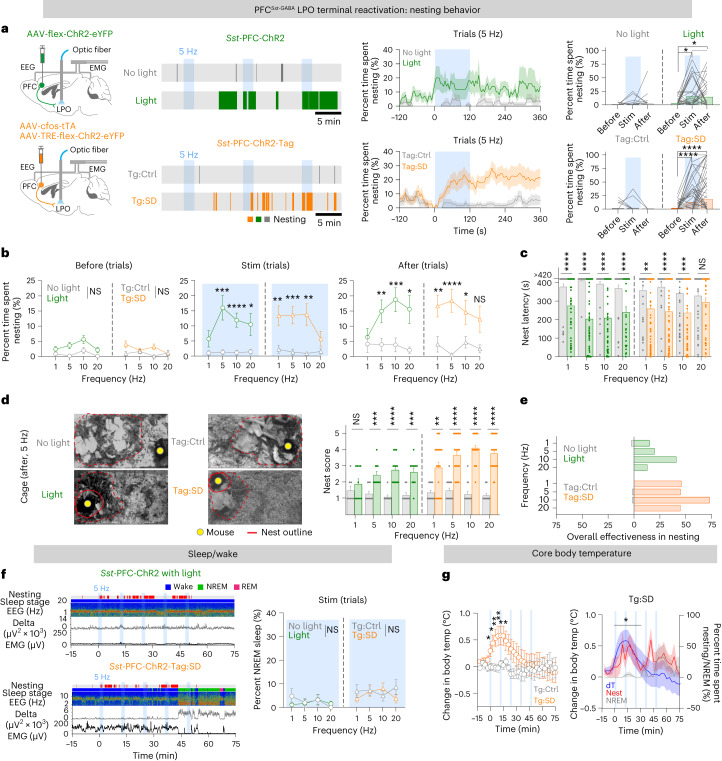
PFC^*Sst*^ projections to the LPO hypothalamus induce nesting. **a**, *Sst*-PFC-ChR2 and *Sst*-PFC-ChR2-Tag:SD mice and optostimulation of PFC^*Sst*^ terminals in the LPO hypothalamus; examples of elicited nesting behavior following stimulation of PFC^*Sst*^ terminals in the LPO hypothalamus of *Sst*-PFC-ChR2 and *Sst*-PFC-ChR2-Tag:SD mice. Gray traces are either no optostimulation (no light, *Sst*-PFC-ChR2 mice) or the ‘on-Dox’ control mice (Tag:Ctrl, *Sst*-PFC-ChR2-Tag:SD mice). Right-hand graph: time course of elicited nesting behavior and statistics of time spent in nesting activity before and after 5-Hz optostimulation trials for 2 min. *Sst*-PFC-ChR2 mice, *n* = 46 trials, *N* = 6 mice (male only); *Sst*-PFC-ChR2-Tag:SD mice, *n* = 75 trials, *N* = 11 mice (six males and five females). Nesting and sleep-start trials were excluded. *Sst*-PFC-ChR2 (light), *P* = 0.0132 (before versus stim), *P* = 0.0126 (before versus after); *Sst*-PFC-ChR2-Tag:SD (Tag:SD), *P* < 0.0001 (before versus stim), *P* < 0.0001 (before versus after) with two-tailed Wilcoxon matched-paired signed-rank test. **b**, Different optostimulation frequencies in the LPO hypothalamus determine nesting activity, during stimulation and after stimulation for *Sst*-PFC-ChR2 and *Sst*-PFC-ChR2-Tag:SD mice and their paired controls (same animal cohort as in **a**). Nesting and sleep-start trials were excluded. *Sst*-PFC-ChR2 (no light versus light): stim, *P* = 0.1202 (1 Hz, *n* = 43 trials), *P* = 0.0004 (5 Hz, *n* = 46 trials), *P* < 0.0001 (10 Hz, *n* = 48 trials), *P* = 0.0374 (20 Hz, *n* = 33 trials); after stim, *P* = 0.009 (5 Hz), *P* = 0.0002 (10 Hz), *P* = 0.033 (20 Hz); *Sst*-PFC-ChR2-Tag:SD (Tag:Ctrl versus Tag:SD): stim, *P* = 0.0055 (1 Hz, *n* = 71 trials), *P* = 0.0002 (5 Hz, *n* = 75 trials), *P* = 0.006 (10 Hz, *n* = 50 trials), *P* = 0.5113 (20 Hz, *n* = 45 trials); after stim, *P* = 0.0051 (1 Hz), *P* < 0.0001 (5 Hz), *P* = 0.0378 (10 Hz), *P* = 0.2751 (20 Hz) with two-sided Mann–Whitney *U*-test. **c**, How optostimulation frequencies in the LPO hypothalamus determine latency to nesting activity during 2-min trials. *Sst*-PFC-ChR2 (no light versus light), *P* < 0.0001 (1 Hz), *P* < 0.0001 (5 Hz), *P* < 0.0001 (10 Hz), *P* < 0.0001 (20 Hz); *Sst*-PFC-ChR2-Tag:SD (Tag:Ctrl versus Tag:SD), *P* = 0.0018 (1 Hz), *P* < 0.0001 (5 Hz), *P* = 0.0006 (10 Hz), *P* = 0.1794 (20 Hz) with two-sided Mann–Whitney *U*-test. **d**, Nest scores. Left: representative nest images after five bouts of 5-Hz stimuli for *Sst*-PFC-ChR2 and *Sst*-PFC-ChR2-Tag:SD mice and their paired control mice. Right: nest scores. *Sst*-PFC-ChR2 (no light versus light), *P* = 0.4577 (1 Hz, *n* = 10 paired sessions), *P* = 0.0003 (5 Hz, *n* = 11 paired sessions), *P* < 0.0001 (10 Hz, *n* = 13 paired sessions), *P* = 0.001 (20 Hz, *n* = 11 paired sessions); *Sst*-PFC-ChR2-Tag:SD (Tag:Ctrl versus Tag:SD), *P* = 0.0024 (1 Hz, *n* = 22 paired sessions), *P* < 0.0001 (5 Hz, *n* = 25 paired sessions), *P* < 0.0001 (10 Hz, *n* = 26 paired sessions), *P* < 0.0001 (20 Hz, *n* = 23 paired sessions) with two-sided Mann–Whitney *U*-test. **e**, Overall effectiveness of optostimulation-evoked nesting activity. Nests scored between 3 and 5 were considered to have successful quality. **f**, Left: example EEG–EMG traces, sleep stage state and aligned nesting activity of *Sst*-PFC-ChR2 and *Sst*-PFC-ChR2-Tag:SD mice during one optostimulus session. Right: how different optostimulation frequencies in the LPO hypothalamus determine time in NREM sleep during 2-min stimuli. **g**, Left: core body temperature change with optostimulation in various frequencies from the baseline time point (*t* = −30 min to 0 min) of *Sst*-PFC-ChR2-Tag:SD mice and their paired controls. *N* = 6 mice (three males and three females). Tag:Ctrl (*n* = 25 sessions) versus Tag:SD (*n* = 20 sessions), *P* = 0.0401 (*t* = −3 min), *P* = 0.0167 (*t* = 0), *P* = 0.0047 (*t* = 3 min), *P* = 0.0041 (*t* = 6 min), *P* = 0.0124 (*t* = 9 min), *P* = 0.0317 (*t* = 12 min) with two-sided Mann–Whitney *U*-test. Right: overlay of time course of percent time spent nesting or in NREM sleep (red and gray, respectively) and change in core body temperature (blue) of *Sst*-PFC-ChR2-Tag:SD mice. Same animals as in **g**. Nest versus NREM, *P* = 3.00 × 10^−2^ (*t* = 0–30 min), two-way RM ANOVA with Bonferroni correction. NS, not significant, *P* ≥ 0.05; *P < 0.05; **P < 0.01; ***P < 0.001; ****P < 0.0001. Mean ± s.e.m. See also Extended Data Fig. 9.

**Extended Data Fig. 9:**
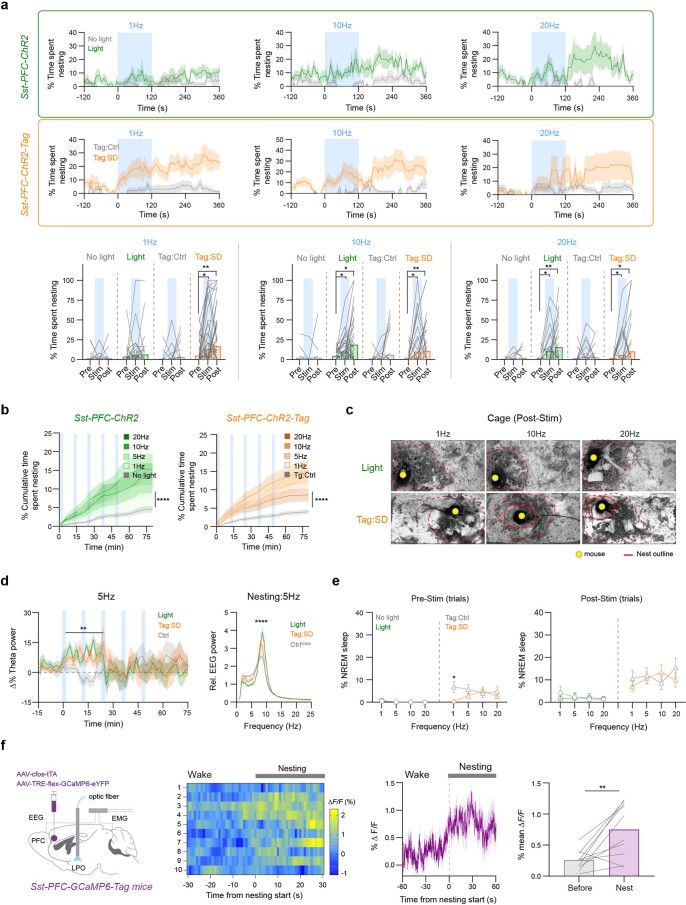
LPO terminal stimulation (Supports Fig. 6). **a**, *Top*: Nesting behavior (% time spent in nesting) before and after 2 min of 1, 10, 20 Hz light stimulation of PFC^*Sst*^ terminals in LPO of *Sst-PFC-ChR2* mice and *Sst-PFC-ChR2-Tag:SD* mice and controls. Bottom: individual statistics for time spent in nesting activity pre-, during, and post- 1, 10, 20 Hz light stimulation in LPO for *Sst-PFC-ChR2* and *Sst-PFC-ChR2-Tag:SD* mice and their controls. Cohorts of *Sst-PFC-ChR2* mice (*N*_*mice*_ = 6), *Sst-PFC-ChR2-Tag* mice (*N*_*mice*_ = 11, (6 males and 5 females)). Nesting/sleep start trials were excluded. *Sst-PFC-ChR2* (Light): Pre versus Stim; *P* = 0.7889 (1 Hz, *n*_*trial*_ = 43), *P* = 0.0183 (10 Hz, *n*_*trial*_ = 42), *P* = 0.0195 (10 Hz, *n*_*trial*_ = 31), Pre versus Post; *P* = 0.8955 (1 Hz), *P* = 0.0129 (10 Hz), *P* = 0.0051 (20 Hz). *Sst-PFC-ChR2-Tag:SD* (Tag:SD): Pre versus Stim; *P* = 0.0103 (1 Hz, *n*_*trial*_ = 71), *P* = 0.0351 (10 Hz, *n*_*trial*_ = 49), *P* = 0.0371 (20 Hz, *n*_*trial*_ = 52), Pre versus Post; *P* = 0.0032 (1 Hz), *P* = 0.0099 (10 Hz), *P* = 0.0161 (20 Hz) with two-tailed Wilcoxon matched-pairs signed rank test. **b**, Cumulative percentage time spent in nesting activity from onset of light stimulus of PFC^*Sst*^ terminals in the LPO of *Sst-PFC-ChR2* mice and *Sst-PFC-ChR2-Tag:SD* mice and their controls. ‘T:0’ indicates the starting point of the opto-stimulation period (blue shading). *Sst-PFC-ChR2* (No light vs. Light); *P* = 2.27 × 10^−3^ (1 Hz, *n*_*session*_ = 11), *P* = 4.86 × 10^−34^ (5 Hz, *n*_*session*_ = 11), *P* = 1.68 × 10^−59^ (10 Hz, *n*_*session*_ = 13), *P* = 9.15 × 10^−32^ (20 Hz, *n*_*session*_ = 10), *Sst-PFC-ChR2-Tag:SD* (Tag:Ctrl vs. Tag:SD); *P* = 4.17 × 10^−89^ (1 Hz, *n*_*session*_ = 24), *P* = 4.79 × 10^−95^ (5 Hz, *n*_*session*_ = 26), *P* = 5.81 × 10^−95^ (10 Hz, *n*_*session*_ = 27), *P* = 2.91 × 20^−22^ (20 Hz,, *n*_*session*_ = 24) with 2-way RM ANOVA with Bonferroni correction. **c**, Nests after a session of 5 bouts of 1, 10, 20 Hz opto-stimulation in *Sst-PFC-ChR2* mice (light) and *Sst-PFC-ChR2-Tag:SD* mice. Yellow dot, position of mouse. Dashed red line, outline of nesting materials; solid red line, nest outline. **d**, Change of theta power from baseline and EEG power spectrum in the nesting state during 5 Hz opto-stimulation in the LPO of *Sst-PFC-ChR2* mice and *Sst-PFC-ChR2-Tag:SD* mice. Change of theta power (T0-30): versus control (No light and Tag:Ctrl), *P* = 9.00 × 10^−3^ (Tag:SD) with Mixed-effects model, EEG power spectrum: *P* = 3.55 × 10^−121^ (Light vs. Ctrl^*Wake*^), *P* = 1.77 × 10^−7^ (Tag:SD vs. Ctrl^*Wake*^) with 2-way RM ANOVA with Bonferroni correction. **e**, Effects of opto-stimulation frequencies in eliciting percent time in NREM sleep, pre- and post-stim per trial for PFC^*Sst*^ terminals in the LPO of *Sst-PFC-ChR2* mice and *Sst-PFC-ChR2-Tag:SD* mice and their controls. Sleep start trials were excluded. *Sst-PFC-ChR2* (No light versus Light): Pre, *P* = 0.75 (1 Hz, *n*_*trial*_ = 45), *P* > 0.9999 (5 Hz, *n*_*trial*_ = 47), *P* > 0.9999 (10 Hz, *n*_*trial*_ = 56), Post, *P* = 0.75 (1 Hz), *P* > 0.9999 (5 Hz), *P* = 0.75 (10 Hz), *P* = 0.625 (20 Hz, *n*_*trial*_ = 49), *Sst-PFC-ChR2-Tag:SD* (Tag:Ctrl vs. Tag:SD): Pre, *P* = 0.0391 (1 Hz, *n*_*trial*_ = 81), *P* = 0.1754 (5 Hz, *n*_*trial*_ = 92), *P* = 0.5742 (10 Hz, *n*_*trial*_ = 66), *P* = 0.4414 (20 Hz, *n*_*trial*_ = 54), Post, *P* = 0.4834 (1 Hz), *P* = 0.4784 (5 Hz), *P* = 0.3657 (10 Hz), *P* = 0.575 (20 Hz) with two-tailed Wilcoxon matched-pairs signed rank. **f**, Calcium photometry signals of PFC^*Sst*^ terminals in LPO of *Sst-PFC-GCaMP6-Tag:SD* mice (*N*_*mice*_ = 3, *n*_*event*_ = 10) during spontaneous nesting, around ZT0. Far right panel: % mean ΔF/F. Before versus nest, *P* = 0.0098 (before vs. nest) two-tailed Wilcoxon matched-paired signed rank test. *P < 0.05, ** *P* < 0.01, *** *P* < 0.001, **** P < 0.0001. *n*_*event*_ = consolidated nesting events. Mean (line) ± SEM (shading) in (**a**), (**b**), (**d**), (**f**). Mean (bar) and individual plot (before-after, line) in (**a**), (**f**). Mean (open circle/triangle) ± SEM (error bar) in (**e**).

We stimulated terminals in the LPO hypothalamus of both constitutively labeled PFC^*Sst*-GABA^ cells (*Sst*-PFC-ChR2 mice) and tagged PFC^*Sst*-GABA^ cells (*Sst*-PFC-ChR2-Tag:SD mice) (Fig. 6a). Starting at ZT 18, terminals were stimulated at 1, 5, 10 and 20 Hz in separate trials (Fig. 6a and Extended Data Fig. 9a), with the same protocol used earlier for stimulating the PFC^*Sst*^ cell soma (Fig. 4a and Extended Data Figs. 1b and 6b). Compared with control mice, all stimulation frequencies induced cumulative nesting behavior (Fig. 6a and Extended Data Fig. 9a,b): during the immediate 2-min optostimulation period, nesting was immediately induced and persisted (Fig. 6a,b and Extended Data Fig. 9a). For both *Sst*-PFC-ChR2 and *Sst*-PFC-ChR2-Tag:SD mice, 5- and 10-Hz stimulation frequencies gave the greatest activation of nesting behavior compared with the pre-stimulation nesting activity (Fig. 6a,b and Extended Data Fig. 9a,b). Similarly, nesting latency (time from stimulus to nesting activity) was shorter in both *Sst*-PFC-ChR2 and *Sst*-PFC-ChR2-Tag:SD mice (Fig. 6c), and nest quality was higher after optostimulation, even more so for *Sst*-PFC-ChR2-Tag:SD mice than for *Sst*-PFC-ChR2 mice, with nesting effectiveness increasing with higher optostimulation frequencies (Fig. 6d,e and Extended Data Fig. 9c). Relative theta power was also increased at the onset of optostimulation (Extended Data Fig. 9d). By contrast, these optostimulations of PFC^*Sst*-GABA^ LPO terminals did not induce NREM sleep above baseline compared with controls (Fig. 6f and Extended Data Fig. 9e). Consistent with these results, the spontaneous calcium activity of tagged PFC^*Sst*-GABA^ LPO terminals in *Sst*-PFC-GCaMP6-Tag:SD mice became elevated during spontaneous nesting (Extended Data Fig. 9f). Optostimulation of tagged PFC^*Sst*-GABA^ LPO terminals increased the core body temperature (Fig. 6g). The core body temperature rose by 1 °C immediately after the first bout of optostimulation, peaking within 10 min of stimulation onset. The temperature profile matched the time course of nesting activity under optostimulation and theta power increase (Fig. 6g and Extended Data Fig. 9d).

**Fig. 7:**
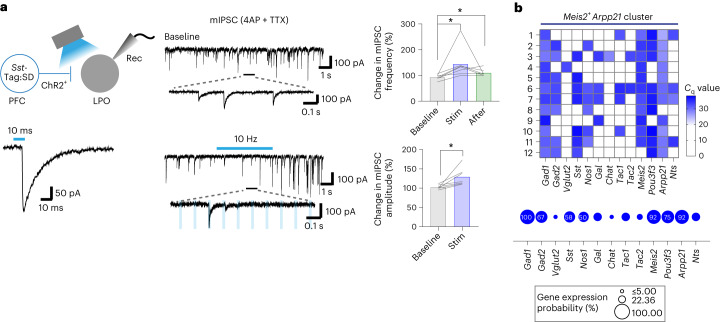
PFC^*Sst*^ projections to the LPO hypothalamus generate inhibitory currents on postsynaptic cells. **a**, mIPSCs from postsynaptic cells in the LPO hypothalamus from *Sst*-PFC-ChR2-Tag:SD mice. Example traces were recorded with 4AP and TTX present for baseline and with 5 s of 10-Hz optostimulation. Each cell shows a different rate of mIPSCs; therefore, we used changes in percent mIPSC occurrence to normalize the changes in mIPSC frequency between conditions (that is, 5 s for before and stimulation and 10 s for after (mean of five trials per cell)). Bar graphs, mean mIPSC frequency changes (baseline versus stim, *P* = 0.0156; baseline versus after, *P* = 0.0312) and mean amplitude changes (baseline versus stim, *P* = 0.0156) with two-tailed Wilcoxon matched-paired signed-rank test. *P < 0.05. Mean (bar) and individual (before–after line). Rec, recording electrode. **b**, Gene expression matrix for LPO cells that responded to stimulating PFC^*Sst*^ terminals. *n* = 12 neurons, *N* = 7 mice.

We looked at the postsynaptic cell types responding to PFC^*Sst*-GABA^ LPO terminal inputs (Fig. 7a). In acute hypothalamic slices containing the LPO hypothalamus, tagged PFC^*Sst*-GABA^ terminals from *Sst*-PFC-ChR2-Tag:SD mice were optostimulated, and then the subsequent mini-inhibitory postsynaptic currents (mIPSCs) were recorded randomly from cell somas adjacent to ChR2-expressing fibers (Fig. 7a). These recordings were made in the presence of action potential blockers (4-aminopyridine (4AP) and tetrodotoxin (TTX)); thus, responses were likely monosynaptically driven. In responding cells, a single 10-ms light pulse induced single mIPSCs (Fig. 7a); on the other hand, optostimulating at 10 Hz (a frequency that evoked nesting) evoked multiple mIPSCs, and their frequency and amplitude increased during optostimulation (Fig. 7a). We then analyzed cytoplasm by multiplex single-cell RT–PCR from cells that had given positive responses (Fig. 7b). All responding cells (12 cells, seven *Sst*-PFC-ChR2-Tag:SD mice) expressed G*ad1*, 92% expressed *Meis2* (encoding Meis homeobox 2) and *Arpp21* (encoding cyclic adenosine monophosphate (cAMP)-regulated phosphoprotein 21), and about 75% expressed *Pou3f3*; about half the cells expressed *Sst* and *Nos1*, and a third expressed *Gal* (galanin) (Fig. 7b). Of these markers, only *Arpp21* expression is reasonably selective, being originally reported as enriched in the LPO hypothalamus compared with other hypothalamic areas^43^.

### PFC^*Sst*-GABA^ terminals in the LH induce sleep but not nesting

**Fig. 8:**
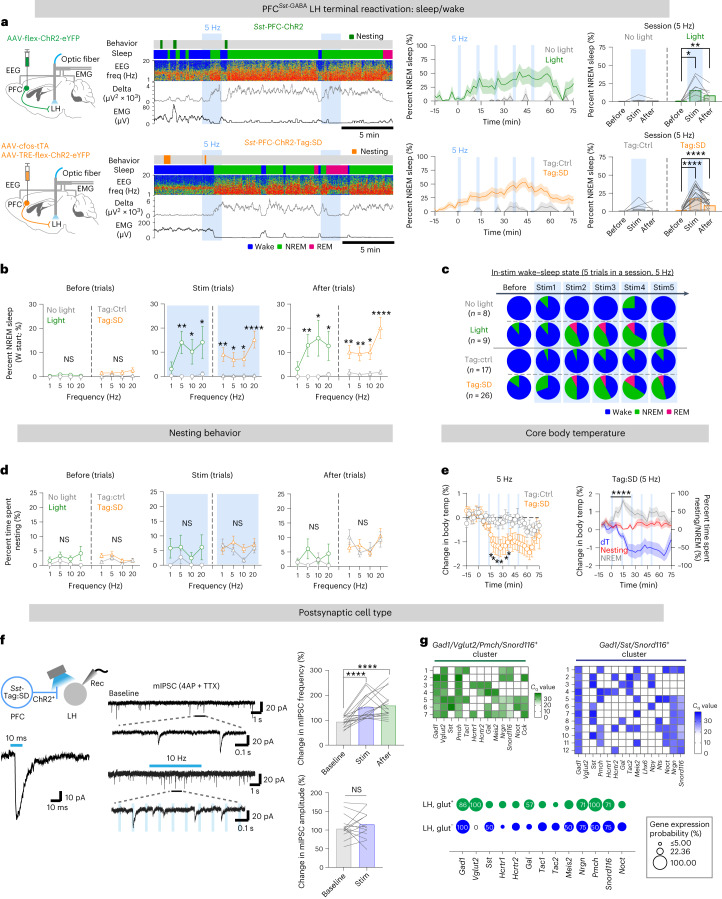
PFC^*Sst*^ projections to the LH induce NREM sleep. **a**, *Sst*-PFC-ChR2 and *Sst*-PFC-ChR2-Tag:SD mice and optostimulation of PFC^*Sst*^ terminals in the LH. Left, EEG–EMG traces, sleep stage and aligned nesting activity following 5-Hz stimulation of PFC^*Sst*^ terminals in the LH of *Sst*-PFC-ChR2 (green, light) and *Sst*-PFC-ChR2-Tag:SD (orange, Tag:SD) mice. Middle: time course of percentage NREM sleep elicited with five bouts of 5-Hz optostimulation. Right: percent NREM sleep before, during and after a session of 5-Hz stimulation. Light, before versus stim (*P* = 0.0313), before versus after (*P* = 0.0078); Tag:SD, before versus stim (*P* < 0.0001), before versus after (*P* < 0.0001) with two-tailed Wilcoxon matched-paired signed-rank test. *Sst*-PFC-ChR2 mice (*N* = 4 mice), *n* = 7 sessions (no light), *n* = 9 sessions (light); *Sst*-PFC-ChR2-Tag:SD mice (*N* = 13 mice, eight males and five females), *n* = 15 sessions (Tag:Ctrl), *n* = 26 sessions (Tag:SD). **b**, The effects of different optostimulation frequencies in the LH in eliciting time spent in NREM sleep during a 2-min stimulus trial. Sleep-start trials were excluded. Stim: light versus no light, *P* = 0.0046 (5 Hz, *n* = 30 trials), *P* = 0.0498 (10 Hz, *n* = 28 trials), *P* = 0.0202 (20 Hz, *n* = 27 trials); Tag:SD versus Tag:Ctrl, *P* = 0.0028 (1 Hz, *n* = 88 trials), *P* = 0.0246 (5 Hz, *n* = 81 trials), *P* = 0.0478 (10 Hz, *n* = 78 trials), *P* < 0.0001 (20 Hz, *n* = 86 trials) with two-sided Mann–Whitney *U*-test. After: light versus no light, *P* = 0.0073 (5 Hz), *P* = 0.0392 (10 Hz), *P* = 0.044 (20 Hz); Tag:SD versus Tag:Ctrl, *P* = 0.0053 (1 Hz), *P* = 0.001 (5 Hz), *P* = 0.0125 (10 Hz), *P* < 0.0001 (20 Hz) with two-sided Mann–Whitney *U*-test. W start, Wake start. **c**, How NREM sleep increases over 5-Hz stimulation trials for optostimulated LH terminals. Same animal cohort as in **b**. *Sst*-PFC-ChR2 mice, *n* = 9 paired sessions; *Sst*-PFC-ChR2-Tag:SD mice, *n* = 26 paired sessions. **d**, Time spent nesting following optostimulation of LH PFC^*Sst*^ terminals. **e**, Left: core body temperature change with optostimulation at various frequencies from the baseline time point (*t* = −30 min to 0 min) of *Sst*-PFC-ChR2-Tag:SD mice and their paired controls. *N* = 6 mice (three males and three females). Tag:Ctrl (*n* = 14 sessions) versus Tag:SD (*n* = 27 sessions), *P* = 0.0186 (*t* = 18 min), *P* = 0.0186 (*t* = 21 min), *P* = 0.0323 (*t* = 24 min), *P* = 0.0131 (*t* = 27 min), *P* = 0.0116 (*t* = 33 min), *P* = 0.0186 (*t* = 36 min) with two-sided Mann–Whitney *U*-test. Right: an overlay of the time course of percent time spent nesting or in NREM sleep (red and gray, respectively) and change in core body temperature (blue) of *Sst*-PFC-ChR2-Tag:SD mice. *P* = 1.503 × 10^−8^ (NREM versus nesting, *t* = 0–30 min) with two-way RM ANOVA. Mean (line) ± s.e.m. (shading). **f**, mIPSCs of postsynaptic LH cells from *Sst*-PFC-ChR2-Tag:SD mice (4AP and TTX were present for baseline, and 5 s of 10-Hz optostimulation was used). Bar graphs: changes in percent mIPSC occurrence to normalize the changes in mIPSC frequency between conditions (that is, 5 s for pre-stimulation and 10 s for post-stimulation (mean of five trials per cell)). Mean mIPSC frequency changes (baseline versus stim, *P* < 0.0001, baseline versus after, *P* < 0.0001) and mean amplitude changes (baseline versus stim, *P* = 0.7609) with two-tailed Wilcoxon matched-paired signed-rank test. *n* = 19 neurons, *N* = 6 mice. **g**, Gene expression matrix for LH cells that responded to optostimulation of PFC^*Sst*^ terminals. *n* = 19 neurons, *N* = 6 mice. glut, glutamatergic. See also Extended Data Fig. 10. NS, not significant, *P* ≥ 0.05; *P < 0.05; **P < 0.01; ****P < 0.0001. Mean (line) ± s.e.m. (shading) in **a**,**e**. Mean (open circle or triangle) ± s.e.m. (error bar) in **b**,**d**,**e**. Individual plots (before–after, line) and mean (bar) in **a**,**f**.

**Extended Data Fig. 10:**
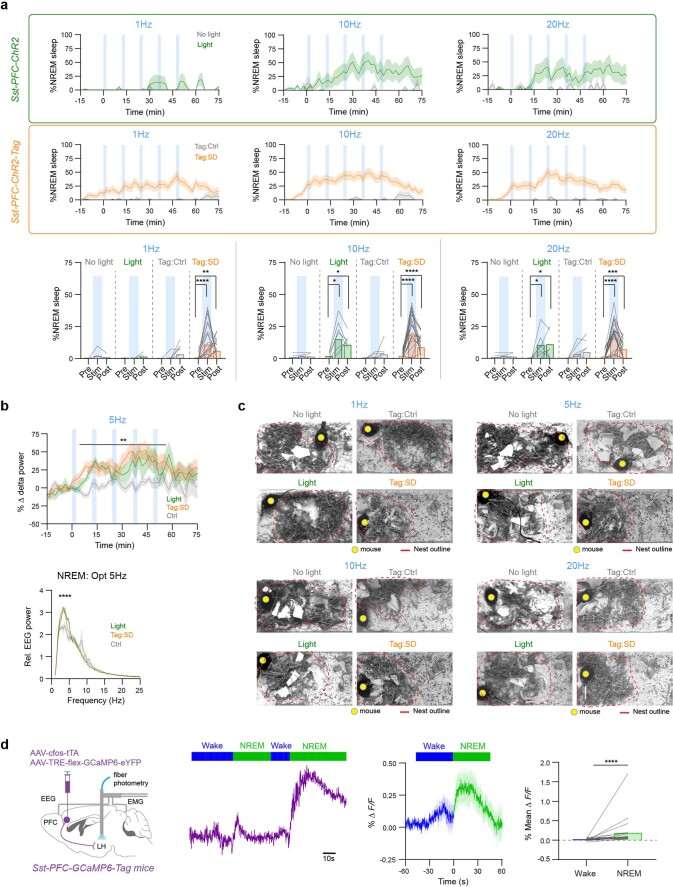
LH terminal stimulation (Supports Fig. 8). **a**, Time course of percent time spent in NREM sleep during sessions of 5 sets of 2 min of 1, 10, or 20 Hz light stimulation of PFC^S*st*^ terminals in the LH in *Sst-PFC-ChR2* mice (*N*_*mice*_ = 4) and *Sst-PFC-ChR2-Tag:SD* mice (*N*_*mice*_ = 13 (8 males and 5 females)) and their controls. Light vs. No light: 10 Hz (n_session_ = 8), *P* = 0.0313 (Pre vs. Stim, Pre vs. Post); 20 Hz (n_session_ = 8), *P* = 0.0156 (Pre vs. Stim), *P* = 0.0234 (Pre vs. Post), Tag:SD vs. Tag:Ctrl:1 Hz (n_session_ = 25), *P* < 0.0001 (Pre vs. Stim), *P* = 0.0017 (Pre vs. Post), 10 Hz (n_session_ = 27), *P* < 0.0001 (Pre vs. Stim, Pre vs. Post), 20 Hz (n_session_ = 27), *P* < 0.0001 (Pre vs. Stim), *P* = 0.0004 (Pre vs. Post) with two-tailed Wilcoxon matched-pairs signed rank. **b**, Change of delta power from baseline and EEG power spectrum in NREM sleep during 5 Hz opto-stimulation in the LPO of *Sst-PFC-ChR2* mice (*n*_*session*_ = 9, *N*_*mice*_ = 4) and *Sst-PFC-ChR2-Tag:SD* mice (*n*_*session*_ = 26, *N*_*mice*_ = 13). *P* = 1.50 × 10^−3^ (Light vs. Ctrl, T0-50), *P* = 7.62 × 10*-3* (Tag:SD vs. Ctrl, T0-50) with Mixed-effects model. For NREM EEG power, *P* = 7.86 × 10^−6^ (Light vs. Ctrl), *P* = 1.34 × 10^−94^ (Tag:SD vs. Ctrl) with 2-way RM ANOVA with Bonferroni correction. **c**, Nests after a session of 5 bouts of 1, 5, 10, 20 Hz opto-stimulation in *Sst-PFC-ChR2* mice (Light) and *Sst-PFC-ChR2-Tag:SD* mice. Yellow dot, position of mouse. Dashed red line, outline of nesting materials. **d**, Calcium photometry signals of PFC^*Sst*^ terminals in LH of *Sst-PFC-GCaMP6-Tag:SD* mice (*n*_*event*_ = 22, *N*_*mice*_ = 3) during transitions between wake and NREM sleep. Mean ΔF/F: *P* < 0.0001 (Wake vs. NREM) with two-tailed Wilcoxon matched-pairs signed rank. *P < 0.05, ** *P* < 0.01, *** *P* < 0.001, **** P < 0.0001. *n*_*event*_ = consolidated NREM sleep. Mean (line) ± SEM (shading) in (**a**), (**b**), (**d**). Mean (bar) and individual plot (before-after, line) in (**a**), (**d**).

As for the LPO experiments, we stimulated terminals in the LH of both constitutively labeled PFC^*Sst*-GABA^ cells (*Sst*-PFC-ChR2 mice) and tagged PFC^*Sst*-GABA^ cells (*Sst*-PFC-ChR2-Tag:SD mice) (Fig. 8a). Starting at ZT 18, terminals were stimulated at 1, 5, 10 and 20 Hz in separate sessions (Fig. 8a and Extended Data Fig. 10a). For both the constitutively labeled PFC^*Sst*-GABA^ terminals and the tagged PFC^*Sst*-GABA^ terminals, all stimulation frequencies induced cumulative NREM sleep but not in control mice or mice with no light stimulation (Fig. 8a and Extended Data Fig. 10a). Sleep induction was cumulative over the 50-min session (with five bouts of optostimulation trials at 10-min intervals) (Fig. 8b,c and Extended Data Fig. 10a). Delta power was increased throughout the optostimulation (Extended Data Fig. 10b). In some instances, rapid eye-movement (REM) sleep was also induced following NREM induction (Fig. 8c). Optostimulation of PFC^*Sst*-GABA^ LH terminals (constitutively labeled or tagged) did not induce nesting behavior (Fig. 8d and Extended Data Fig. 10c). Consistently, the spontaneous calcium activity of tagged PFC^*Sst*-GABA^ LH terminals in *Sst*-PFC-GCaMP6-Tag:SD mice became elevated during the first part of NREM sleep (Extended Data Fig. 10d). Furthermore, the mean core body temperature started to decrease as soon as the first bout of optostimulation was delivered to *Sst*-PFC-ChR2-Tag:SD mice (Fig. 8e). This coincided with the evoked NREM induction. The lowered temperature was sustained while animals were in NREM sleep (Fig. 8e). Overall, these results show that selective stimulation of PFC^*Sst*-GABA^ terminals in the LH, whether activity tagged with ChR2 or constitutively labeled with ChR2, can initiate NREM sleep and lower core body temperature.

We examined in acute LH slices the postsynaptic cell types that respond to PFC^*Sst*-GABA^ terminals (Fig. 8f). Tagged *Sst*-expressing terminals from *Sst*-PFC-ChR2-Tag:SD mice were optostimulated, and mIPSCs were recorded from cell somas close to ChR2-expressing fibers (Fig. 8f). In responding cells (19 cells, six *Sst*-PFC-ChR2-Tag:SD mice), a single 10-ms light pulse induced single mIPSCs (Fig. 8f); optostimulating at 10 Hz evoked multiple mIPSCs; during stimulation, the frequency but not the mean amplitude of mIPSCs increased (Fig. 8f). We analyzed the cytoplasm of cells that responded by single-cell multiplex RT–qPCR (Fig. 8g). Nearly all cells expressed *Gad1* (18 cells), and 37% (seven cells) of these expressed *Slc17a6* (*Vglut2*). Of these, all *Vglut2*-expressing cells expressed *Pmch*; *Gal* expression was preferentially associated with *Gad1*-, *Vglut2*- and *Pmch*-expressing cells. For the *Gad1*-expressing cells that did not express *Vglut2*, about half expressed *Sst*. Many responding cells expressed *Snord116* and *Nrgn* (Fig. 8g). Overall, a wide range of cells in the LH were inhibited by PFC^*Sst*-GABA^ terminals.

## Discussion

The PFC enables mammals to respond to situations, including internal states, with appropriate planning and actions^35^. We hypothesized that ‘tiredness’ is an internal state, and, indeed, the PFC seems particularly sensitive to fatigue^28,29,31^. An appropriate response to tiredness for mice might be nest building and sleeping. The PFC executes its planning role by sending projections to the hypothalamus and other subcortical areas^35,37,44^. We showed that, when mice (male or female) are deprived of sleep, their post-SD sleep-preparatory behavior (nesting), elevated body temperature and concurrent elevated EEG theta power and then, subsequently, RS (sleep with higher delta power^41^) and lower body temperature are elicited by PFC^*Sst*-GABA^ cells projecting to the ventral LPO hypothalamus and the ventral LH, respectively. Tagged PFC^*Sst*-GABA^ terminals had enhanced calcium activity during nesting and sleep, respectively, and induced fast inhibitory postsynaptic currents (IPSCs) on target cells. Activity tagging enabled us to identify PFC^*Sst*-GABA^ cells with specific properties: cells that would have been difficult to capture without using the c-Fos system. However, opto-activation of PFC^*Sst*-GABA^ terminals with constitutively expressed ChR2 in the LPO hypothalamus and the LH also caused nesting, body temperature changes and NREM sleep, suggesting that these PFC cells likely contribute to baseline sleep-preparatory behavior, temperature regulation and NREM sleep as well. The PFC also might feature in deciding where to sleep even without excessive fatigue.

Many neocortical *Sst*-expressing GABA cells are slow spiking and primarily innervate dendrites of pyramidal cells^42,45^. About 70% of the PFC^*Sst*-GABA^ cells captured by activity-tagging behaviors are different: they are more excitable and fast spiking (up to 20 Hz) and send long-range projections. All stimulation frequencies of PFC^*Sst*-GABA^ terminals in the LPO hypothalamus and the LH produced nesting behavior and NREM sleep, although there was some drop off in elicited behavior at 20 Hz. Recently, around 16 subtypes of mouse neocortical *Sst*-expressing cells have been identified^42^, and one is fast spiking and coexpresses *Parv* (*Pvalb*) but not *Nos1*, partially matching the PFC^*Sst*-GABA^ cells. It is also possible that, rather than discreet subtypes, there is a continuum of *Sst*-expressing cells^46^, and that, in this study, we enriched for the fast-spiking end of the spectrum.

Although direct optostimulation of PFC^*Sst*-GABA^ terminals in the LH induced immediate NREM sleep and lower body temperature, the mechanism that underlies initial nesting with raised body temperature, followed by delayed sleep with lower temperature remains unclear. Several types of PFC^*Sst*-GABA^ cells might be responsible. These cells might have been tagged at different time points during the procedure, for example, SD versus nesting versus RS, although it is striking that the majority of tagged *Sst*-expressing cells were the unusual, fast-spiking type and only 30% were the slow-spiking type. A limitation of our approach is that the kinetics of the Dox system do not allow the different segments of mouse behavior (sustained wakefulness, nesting, RS) to be cleanly segregated. Another limitation is that we did not examine the potential role of co-released SST peptide from PFC^*Sst*-GABA^ cells; for example, SST release might lead to gradual behavioral changes and might contribute to delayed sleep effects in the natural setting.

We do not know what activates PFC^*Sst*-GABA^ cells (which are relatively easy to excite) during SD–nesting–RS behaviors. PFC^*Sst*-GABA^ cells might be activated indirectly by decreased activity in dopaminergic PFC-projecting VTA neurons, as decreasing VTA dopamine activity induces nesting^1^. Intriguingly, many neocortical cells beyond the PFC were captured during the tagging procedure, including *Sst*-expressing cells in the VC. However, VC^*Sst*^ cells did not induce nesting or sleep when reactivated and did not send axons to the hypothalamus; therefore, the role of these cells in this context is unclear. Similarly, although neocortical *Nos1*-expressing cells are activated during RS^25–27^, reactivating tagged PFC^*Nos1*^ cells did not stimulate nesting, only transiently induced NREM sleep and did not change body temperature. A rare type of neocortical *Nos1*-positive, *Sst*-negative GABAergic cell is active in the downstate of NREM sleep but does not contribute to sleep^47^.

How might the PFC^*Sst*-GABA^-to-LPO hypothalamus connection induce nesting, enhance theta power and raise body temperature? In the LPO hypothalamus, cells inhibited by PFC^*Sst*-GABA^ terminals were all GABAergic and nearly all expressed *Arpp21*, a gene encoding a cAMP-regulated phosphoprotein that binds microRNA^48^. Many cells also expressed *Nos1* and *Sst*, and about a third expressed the transcript for galanin. More work is needed to identify how the inhibited *Arpp21*-expressing cells interact with the other cells or regions that regulate nesting before sleep^7^. Activating LPO galanin-expressing cells lowers body temperature^40,49^, whereas lesioning or removing them increases body temperature^40^. Thus, the PFC^*SSt*-GABA^-to-LPO hypothalamus connection might increase body temperature by inhibiting galanin neurons. There are likely parallel routes by which nesting and sleep induction reinforce one another. Nesting provides thermal microclimates, warming the skin; this in turn, via *Nos1*-, *Vglut2*-expressing cells in the MPO hypothalamus, promotes NREM sleep and concomitant body cooling when sleep starts^3,9,50^.

How do PFC^*Sst*-GABA^-to-LH connections induce NREM sleep? VTA^*Sst*-GABA^ neurons, which also project to the LH, also induce sleep^51–53^, suggesting that GABA projections to the LH could be a common mechanism for sleep induction. Some LH GABA cells induce wakefulness when activated^54–56^, and, if these are targeted by PFC^*Sst*-GABA^ (and VTA^*Sst*-GABA^) terminals, this could produce NREM sleep. The LH cells that responded with evoked IPSCs from PFC^*Sst*-GABA^ terminals were GABA cells that often coexpressed *Vglut2* and *Pmch*. Some glutamatergic *Pmch* cells in the LH project to and excite septal GABA cells^57^; in principle, glutamatergic *Pmch*-expressing cells could also excite the wake-promoting LH GABA cells. Similar to the MPO hypothalamic *Nos1*-expressing, glutamate cells that co-regulate NREM induction and decreases in body temperature^9^, activating PFC^*Sst*-GABA^ terminals in the LH acutely decreased body temperature, but we do not know the responsible cells.

In summary, our findings indicate that the PFC issues top–down instructions to the hypothalamus to regulate both behavioral preparation for sleep (nesting and increased body temperature) and activation of sleep-induction circuitry that induces NREM sleep (and associated lower body temperature), ensuring that optimal sleep takes place in a suitable place.

## Methods

### Mice

All experiments were performed in accordance with the UK Home Office Animal Procedures Act (1986) and approved by Imperial College’s Animal Welfare and Ethical Review Body. The following types of mice were used: *Vgat-ires-Cre* (*Slc32a1*^*tm2(cre)Lowl*^/J) mice (Jackson Laboratory stock 016962), kindly provided by B. B. Lowell (Beth Israel Deaconess Medical Center & Harvard Medical School, USA)^58^; *Nos1-ires-Cre*^*tm1(cre)Mgmj*^/J mice (Jackson Laboratory stock 017526), kindly provided by M. G. Myers (University of Michigan, Ann Arbor, USA)^59^; *Sst-ires-Cre* (*Sst*^*tm2.1(cre)Zjh*^/J) mice (Jackson Laboratory stock 013044), kindly provided by Z. J. Huang (Cold Spring Harbor Laboratory, Cold Spring Harbor, USA)^60^; and C57BL/6J mice (supplied by Charles River). For all experiments using *Sst-ires-Cre* mice, both male and female mice were used. All mice were congenic on the C57BL/6J background. Mice were maintained on a 12-h–12-h light–dark cycle at constant temperature and humidity with ad libitum food and water.

### AAV transgene plasmids and AAV preparation

We have described most of the plasmids containing adeno-associated virus (AAV) transgenes previously: *pAAV-cFos-tTA-pA* (Addgene plasmid 66794)^38^, *pAAV-P*_*TRE-tight*_*-flex-hM3Dq-mCherry* (Addgene plasmid 115161)^9^, *pAAV-P*_*TRE-tight*_*-flex-ChR2-EYFP* (Addgene plasmid 183765)^39^, *pAAV-P*_*TRE-tight*_*-flex-GCaMP6-EYFP* (Addgene plasmid 183809)^39^ and *pAAV-flex-EGFP*^61^. Plasmid *pAAV-EF1α-flex-hChR2(H314R)-EYFP* was a gift from K. Deisseroth (James H. Clark Center, Stanford University Medical School, Stanford University, USA) (Addgene plasmid 20298). To generate *pAAV-P*_*TRE-tight*_*-flex-ChR2-mCherry*, an NdeI site was introduced between the ChR2- and EYFP-coding segments of *pAAV-P*_*TRE-tight*_*-flex-ChR2-EYFP*. This new mutated construct was digested with NdeI and AscI to remove the EYFP-coding segment, and the rest of the construct (5.6-kb band) was gel purified. Using a plasmid containing the mCherry-coding gene as template, the mCherry reading frame was amplified by PCR from just before the start codon with the forward primer and with an AscI site just after the stop codon for the reverse primer. This PCR product was digested with NdeI and AscI and ligated with the 5.6-kb fragment previously mentioned to give *pAAV-P*_*TRE-tight*_*-flex-ChR2-mCherry*. For the *pAAV-EF1a-flex-hChR2(H134R)-mCherry* plasmid, an NdeI restriction site was introduced by mutagenesis into *pAAV-EF1a-flex-hChR2(H134R)-EYFP* (Addgene, 20298), between the ChR2 and EYFP reading frames, keeping the correct reading frame. The new mutated plasmid was double digested with AscI and NdeI to remove the EYFP-coding fragment, and the remaining 6.5-kb DNA band, *AAV-EF1a-flex-ChR2(H134R)*, was gel purified. An mCherry reading frame was amplified by PCR, introducing an NdeI site before the start codon of mCherry and an AscI site after the stop codon. This PCR product was double digested with AscI and NdeI and ligated into the double-digested (AscI and NdeI) AAV-EF1a-flex-ChR2(H134R) fragment.

The AAV was a mixed capsid serotype (AAV1 and AAV2). To produce AAVs, the adenovirus helper plasmid *pFΔ6* and the AAV helper plasmids *pH21* (AAV1) and *pRVI* (AAV2) and the *pAAV* transgene plasmids were all co-transfected into HEK293 cells, and the subsequent AAV particles were collected on heparin columns^62^. This was done in house. The virus titer dilutions and volumes used for each experiment are listed in Supplementary Tables 1 and 2.

### Stereotaxic surgery

One week before surgery, mice were placed on 200 mg per kg Dox-containing (Envigo, TD.09265) chow. Stereotaxic virus injections were performed using an Angle Two apparatus (Leica) linked to a digital brain atlas (Leica Biosystems) and a stainless steel 33-gauge, 15-mm, PST3 internal cannula (Hamilton) attached to a glass syringe (10-µl, Hamilton, 701). Unless otherwise specified, virus was bilaterally injected at 0.1 µl min^−1^, with two injections per site of 0.25 µl for in vivo and electrophysiology experiments and 0.35 µl for cell counting and axonal tracing experiments. The injection coordinates were as follows: PFC, mediolateral (ML) (±0.4 mm), anteroposterior (AP) (2.1 mm), dorsoventral (DV) (−2.45 mm); and VC, ML (±2.38 mm), AP (−2.54 mm), DV (−0.92 mm). For optogenetic and photometry experiments, we first injected the AAV mixture unilaterally (for PFC and VC) and bilaterally (for LPO hypothalamus and LH) and then implanted a monofiber optic cannula (I.D., 200 µm; 0.37 NA; Thorlabs, FT200EMT) unilaterally directly above the following coordinates: LH, ML (±1.0 mm), AP (−1.56 mm), DV (5.16 mm); and LPO, ML (±0.75 mm), AP (0.40 mm), DV (5.15 mm).

For sleep recordings, two electromyography (EMG) wire electrodes were inserted in the neck extensor muscles and two EEG screw electrodes were placed at ML (−1.5 mm), AP (+1.5 mm) and ML (−1.5 mm), AP (−2.0 mm) relative to the bregma. A third EEG electrode was placed at ML (+1.5 mm), AP (−2.0 mm) for optogenetic recording. All instrumented mice were housed singly to avoid damage to the head stage and were allowed to recover and, for the viral transgenes, to adequately express for at least 3 weeks.

### Activity-tagging behavioral protocols and controls

This was carried out similarly to how we did this previously^9,38,39^. Two AAVs, *AAV-P*_*cFos*_*-tTA* and *AAV*-*P*_*TRE-tight*_*-flex-‘effector gene’* (for example, *ChR2-EYFP*, h*M3Dq-mCherry*), were bilaterally co-injected into the PFC of *Vgat*^Cre^ or *Nos1*^Cre^ or *Sst*^Cre^ mice. To repress the activity-tagging system, mice were maintained on Dox-containing chow for 1 week before the surgery and at least 3 weeks after the surgery. Before SD, mice were taken off Dox for 2 d and then deprived of sleep for 5 h by introducing new objects, beginning in the new cage at the start of the ‘lights-on’ (ZT 0) period. Mice were gently placed back into their home cages with Dox-containing chow and allowed RS. Mice were habituated to Neurologger 2A EEG recording devices for at least 2 d before SD and RS were performed. During this time, a 24-h EEG–EMG baseline recording was obtained, and SD and RS were monitored and confirmed offline. Any mice that failed to show 5 h of clear SD and an RS accompanied by a delta power increase were discounted from the chemogenetic or optogenetic experiments.

Singly housed mice were kept in their home cage with Dox-containing chow before and after SD. Optostimulation was carried out in the home cage, but any existing nest was destroyed, and the material was mixed with new nesting materials to reduce the habituation period. Food was purposely placed closer to the water bottle and away from the nesting materials to segregate nest-building behavior from food- and water-seeking behaviors (Extended Data Fig. 1).

### EEG–EMG recordings and analysis

EEG and EMG signals were recorded using Neurologger 2A devices^63^ at a sampling rate of 200 Hz, and the data were visualized with Spike2 software (Cambridge Electronic Design). EEG signals were high-pass filtered offline at 0.5 Hz (−3 dB), and EMG signals were bandpass filtered offline at 5–45 Hz (−3 dB). To define the vigilance states of wake and NREM and REM sleep, delta power (0.5–4.5 Hz), theta power (5–10 Hz) and theta/delta (T:D) ratios were calculated. Automated sleep scoring was performed using a Spike2 script, and the results were manually corrected.

### Chemogenetics

Mice were allowed to habituate to the Neurologger 2A devices minimally 2 d before SD and RS were performed. Two days after SD, CNO (4936, Tocris, dissolved in saline, 1 mg per kg and 5 mg per kg) or saline was injected i.p. at ZT 18 (that is, mid-‘lights-off’ period, that is, at the time when mice were most active and least likely to build a nest or sleep), and vigilance states were recorded. Mice were split into random groups that received either saline (day 1) and CNO (day 2) or CNO (day 1) and saline (day 2) injections at the same circadian time (Extended Data Fig. 1c). Mice were habituated again to the Neurologger 2A devices at least 1 h before ZT 18 (i.p. injection, *t* = 0).

#### Consideration of clozapine-*N*-oxide doses

The effects of different CNO doses (1, 5 and 10 mg per kg, injected i.p.) on sleep have been systematically tested in wild-type (C57BL/6J) mice that do not express hM3Dq receptors^64^. The injections were given at a time when mice were most sleepy, the beginning of the ‘lights-on’ period^64^. In the first 2 h following CNO injection, there was no significant main effect on the proportion of time spent awake or in NREM sleep or REM sleep^64^. For NREM sleep, there was no consistent effect of CNO dose on sleep latency, but, at 5 and 10 mg per kg CNO, there was a small but significant effect of prolonging individual NREM episodes and reducing their number, so that sleep architecture was slightly changed, but NREM sleep amount was unchanged. Thus, for our study, when we gave CNO during the ‘lights-off’ phase when mice were most awake, the difference between 1 and 5 mg per kg CNO is not likely to cause any background effects on sleep–wake dynamics.

### Optogenetics

Mice were allowed to habituate to the Neurologger 2A devices minimally 2 d before SD and RS were performed. Optogenetic stimulations were generated by a 473-nm diode-pumped solid-state laser with a fiber coupler (Shanghai Laser, BL473T3T8U-100FC, Shanghai Laser & Optics Century) or a 465-nm Doric Connectorized LED (CLED_465, Doric Lenses). Stimulation protocols were programmed and controlled using Signal software (Cambridge Electronic Design) and Micro1401 (CED) for the laser and Doric Neuroscience Studio version 5.3.3.14 (Doric Lenses). Laser and LED power was kept in the range of 2–5 mW at the tip of the optic fiber (0.8–1.0 mW mm^−2^ at a depth of 1 mm) unless stated otherwise.

Optostimulation was carried out during ZT 18 (the mid-‘lights-off’ period in the animal house). Before starting the stimulation protocol, all mice were habituated for at least 30 min to the environment. For controls for *Vgat*-PFC-ChR2-Tag:SD and *Vgat*-VC-ChR2-Tag:SD mice, we used *Vgat*-PFC-GFP mice (AAV-flex-EGFP was injected into the PFC of *Vgat*^Cre^ mice) and *Vgat*-PFC-ChR2-Tag mice that had had Dox removed from their diet for the same time duration as the paired experimental cohorts but that had not been deprived of sleep (*Vgat*-PFC-ChR2-Tag:Ctrl mice). Results from both groups of controls were pooled. For the control for *Sst*-PFC-ChR2-Tag:SD mice, we used the same *Sst*-PFC-ChR2-Tag:SD mice before the tagging procedure (*Sst*-PFC-ChR2-Tag:Ctrl mice). For controls for *Sst*-PFC-ChR2 mice, we gave no-laser or low-power (0.5–1 mW at the tip, 0.1–0.15 mW mm^−2^ at a depth of 1 mm) optostimulation to the same *Sst*-PFC-ChR2 mice.

### Calcium photometry

This was performed as described previously^65^. Mice were allowed to habituate to Neurologger 2A devices minimally 2 d before SD and RS were performed. Light was generated by a 473-nm diode-pumped solid-state laser with a fiber coupler (Shanghai Laser, BL473T3T8U-100FC, Shanghai Laser & Optics Century) or a 465-nm Doric Connectorized LED (CLED_465, Doric Lenses). Laser and LED power was kept in the range of 70–90 µW at the tip of the optic fiber (0.22–0.30 mW mm^−2^ at maximum). The GCaMP6 output was filtered at 500–550 nm through the fluorescence cube, converted to voltage by a photodiode and then amplified by the lock-in amplifier (SR810, Stanford Research Systems) with a time constant of 30 ms. Photometry, EEG and EMG data were aligned offline using Spike2 software and analyzed using custom MATLAB scripts. For each experiment, the photometry signal *F* was normalized to the baseline using the function Δ*F*/*F* = (*F* − *F*_0_)/*F*_0_, where *F*_0_ is the mean fluorescence across the signal analyzed. The baseline photometry values for photobleaching and photometry signal drift during long recording were corrected with a custom MATLAB script.

### Core body temperature recording

Core body temperature was recorded using temperature loggers (DST nano, Star-Oddi) implanted abdominally as described previously^9^. A pre-defined program was set to sample the temperature data every 3 min for baseline core body temperature and during chemogenetic and optogenetic experiments. At the end of the experiments, the loggers were retrieved, and the data were downloaded and analyzed offline. For delta change against baseline analysis, the mean 24-h baseline body temperature was taken from 5 consecutive days of recording before the experimental period.

### Behavioral analysis and nest scoring

All behavior was monitored with a video camera, which was placed above the test cage, and analyzed offline after the experiments. All evaluation was carried out on pre-blinded recording data by more than one experimenter. The difference was reviewed and corrected before unblinding. Videos were synchronized with stimulation protocols. Video nesting behavior over time was scored using Behavioral Observation Research Interactive Software (BORIS)^66^ and aligned with sleep scoring in Spike2. Nesting behavior was defined as pushing and carrying the nesting material; or fluffing the material up or body wriggling at the center of the nest site and making space for the new nesting material.

Before the initial habituation period (starting at ZT 17) of optogenetic experiments, all previously existing nest material was removed from the home cage of the test mice, and we placed 8 g of a mixture of old and new shredding papers away from food and water. The baseline nest condition was remotely checked 5–10 min before ZT 18 (optostimulation, *t* = 0) without disturbing the test mice. For chemogenetic experiments, the cage was prepared 30 min to 1 h before i.p. injection at ZT 18 and monitored with an overhead video camera for 5 h.

We evaluated nest scores offline by adapting a five-point scale^1,67,68^: (1) nest materials are not noticeably touched (<10% change from baseline), (2) nest materials are partially gathered (10–50% change from baseline), (3) nest materials are sorted and gathered, but some are spread around the cage (50–90% change from baseline), (4) nest materials are sorted and gathered; identifiable but flat, (5) a perfect or near-perfect nest with a crater.

Nesting effectiveness during an optostimulation session was calculated by multiplying availability (percent, overall nesting time ÷ 25% of overall session time), performance (100%, by assuming that all scored nesting behaviors contribute to nest building) and nest quality (percent, ‘good’ quality nest (nest score 3–5) ÷ overall nest).

### Immunohistochemistry and imaging

Mice were transcardially perfused with 4% paraformaldehyde (Thermo Scientific) in PBS. Brains were removed, and 35-μm-thick coronal sections (unless otherwise specified) were cut using a Leica SM2010R microtome or a Thermo Scientific HM 450 Sliding Microtome. Free-floating sections were rinsed once with PBS and processed for epitope retrieval by incubating sections in 0.05% Tween-20 in 10 mM sodium citrate buffer (pH 6.0) at 80–85 °C for 30 min. Sections were allowed to cool down to room temperature and then washed three times with PBS for 10 min. Sections were blocked with a solution of 20% goat serum (NGS, Vector), 0.2% Triton X-100 and PBS for 1 h at room temperature and incubated with primary antibody at an adequate dilution in 2% NGS, 0.2% Triton X-100, PBS solution overnight at 4 °C. Incubated slices were washed three times with PBS for 10 min at room temperature and incubated with a secondary antibody (Molecular Probes) at an adequate dilution in 2% NGS, 0.2% Triton X-100, PBS solution for 1.5 h at room temperature. Slices were washed three times with PBS for 10 min at room temperature and incubated with Hoechst 33342 (Life Technologies) at 1:5,000 in PBS for up to 10 min at room temperature. After a double wash with PBS, slices were mounted on slides with ProLong Gold Antifade Reagent (Invitrogen). Primary antibodies were rabbit anti-GFP (Invitrogen, A6455, 1:1,000), chicken anti-GFP (Abcam, ab13970, 1:1,000), mouse anti-mCherry (Clontech, 632543, 1:1,000), rabbit polyclonal c-Fos (Santa Cruz Biotechnology, sc-52, 1:4,000) and mouse monoclonal Gad67 (Millipore, MAB5406, 1:500). Secondary antibodies were Alexa Fluor 488 goat anti-chicken (Invitrogen, A11039, 1:500), Alexa Fluor 488 goat anti-rabbit (Invitrogen, A11008, 1:500) and Alexa Fluor 594 goat anti-mouse (Invitrogen, A11005, 1:500). Images were taken with an Axiovert 200M inverted widefield microscope (Zeiss) and Leica SP8 inverted confocal microscopes. Images were analyzed and merged, and scale bars were added using Fiji version 2.9.0. All final figures were assembled using Adobe Illustrator version 27.5.

### Acute brain slice preparation

*Vgat*-PFC-ChR2-Tag and *Sst*-PFC-ChR2-Tag mice that had undergone the tagging protocol and that had been placed back onto Dox for a minimum of 1 d were euthanized by cervical dislocation. Age-matched *Sst*-PFC-ChR2 mice were euthanized at the same time point after AAV-injection surgery without tagging. The brain was quickly removed and placed into cold oxygenated *N*-methyl-d-glucamine solution (in mM): 93 *N*-methyl-d-glucamine, 93 HCl, 2.5 KCl, 1.2 NaH_2_PO_4_, 30 NaHCO_3_, 20 HEPES, 25 glucose, 5 sodium ascorbate, 2 thiourea, 3 sodium pyruvate, 10 MgSO_4_, 0.5 CaCl_2_. Coronal brain slices (250-μm thickness) encompassing the PFC, the LPO hypothalamus and the LH were obtained using a vibratome (Vibrating Microtome 7000smz-2, Campden Instruments). Slices were transferred to a submersion chamber and continuously perfused at a rate of 1–2 ml min^−1^ with fully oxygenated aCSF (in mM): 120 NaCl, 3.5 KCl, 1.25 NaH_2_PO_4_, 25 NaHCO_3_, 10 glucose, 2 CaCl_2_, 1 MgCl_2_. *Sst*^ChR2:Tag:SD^ and *Sst*^ChR2^ neurons were identified by their EYFP or mCherry signal under fluorescence illumination (LED4D, Thorlabs, coupled to a YFP or mCherry excitation filter).

### Ex vivo electrophysiology

Acute brain slices were transferred to a slice-recording chamber (Scientifica) and were continuously perfused at a rate of 3–5 ml min^−1^ with fully oxygenated aCSF at room temperature. Whole-cell patch-clamp recordings were performed with a MultiClamp 700B amplifier and a 1440A interface (Molecular Devices). Data were measured using Clampfit version 10.7 software (Molecular Devices). A 470-nm blue light was delivered by a TTL-controlled LED (LED4D067, Thorlabs) directed through the objective (×40 water-immersion lens) with a light intensity of ~2 mW. Fluorescent cells were visualized and illuminated with an LED lamp. Data were collected 2 min after obtaining a stable whole-cell configuration. Access and input resistances were monitored throughout the experiments using a 5-mV voltage step. The access resistance was typically <20 MΩ, and results were discarded if resistance changed by more than 20%. Membrane capacitance (*C*_m_) was measured under voltage clamp at −50 mV using a hyperpolarizing 10-mV, 250-ms step. Neurobiotin (0.1%) was included in the intracellular solutions to identify the cell position and morphology following recording.

To obtain the data shown in Fig. 5 and Extended Data Figs. 7 and 8, the current-clamp mode was used for recording intrinsic membrane properties, with electrodes (4–6 MΩ) filled with an internal solution containing the following (in mM): 140 potassium gluconate, 5 KCl, 10 HEPES, 0.1 EGTA, 2 MgCl_2_, 2 NaATP and 0.2 NaGTP, pH 7.3 (280–285 mOsm). Under current-clamp mode, a ramp depolarization of 100 mV s^−1^ (20-mV increments) and a series of 12 (1-s duration) 20-mV voltage steps of increasing amplitude from −40 mV to 200 mV were injected to evoke action potentials to observe cell excitability. Light-evoked action potentials were obtained with a single light pulse of 10 ms (interval, 30 s), or a set frequency of multiple 10-ms light pulses for 5 s (1, 5, 10, 20 Hz; interval, 1 min) were given to mimic the optic stimulation in behavior and/or sleep experiments.

For the data in Figs. 5 and 6, the recorded neurons were visually selected from cells immediately adjacent to EYFP or mCherry fluorescence signals. The membrane potential was held at −70 mV, with electrodes (3–5 MΩ) filled with (in mM) 125 KCl, 20 NaCl, 10 HEPES, 1 EGTA, 0.3 CaCl_2_, 1 MgCl_2_, 2 NaATP and 0.5 NaGTP, pH 7.3 (280–285 mOsm). NBQX (25 μM) and d-AP5 (50 μM) were added to recording aCSF solution to block AMPAR- and NMDAR-mediated glutamate responses. Light stimulation was given 5 min after obtaining a stable whole-cell configuration. Light-evoked monosynaptic mIPSCs were recorded in the presence of 1 μM TTX and 100 µM 4AP. Light-evoked IPSCs were obtained with a single light pulse of 10 ms (interval, 30 s), or a set frequency of multiple 10-ms light pulses for 5 s (1, 5, 10, 20 Hz; interval, 1 min) were given to mimic the optic stimulation in behavior and/or sleep experiments. Frequency, amplitude and decay time constants of mIPSCs were analyzed offline with MiniAnalysis (Synaptosoft).

Cytoplasmic contents of recorded neurons were aspirated into recording electrodes and expelled into cell lysis or DNase I solution for the single-cell RT–PCR assay, and recorded brain slices were fixed in 4% PFA for further immunostaining to confirm the anatomical location of recorded neurons and their morphology.

### Single-cell multiplex RT–qPCR

cDNA synthesis was performed using the Single Cell-to-CT Kit (Invitrogen), and multiplex qPCR was performed using the TaqMan Gene Expression Assay system (Applied Biosystems). All TaqMan probes were purchased from Applied Biosystems and are as follows: *Arpp21* (Mm00473630_m1), *Cck* (Mm00446170_m1), *Chat* (Mm01221880_m1), *Chodl* (Mm00507273_m1), *Crhbp* (Mm01283832_m1), *Dlx1* (Mm00438424_m1), *Gad1* (Mm04207432_g1), *Gad2* (Mm00484623_m1), *Gal* (Mm00439056_m1), *Hcrtr1* (Mm01185776_m1), *Hcrtr2* (Mm01179312_m1), *Htr3a* (Mm00442874_m1), *Lhx6* (Mm01333348_m1), *Meis2* (Mm00487748_m1), *Noct* (Mm00802276_m1), *Nos1* (Mm01208059_m1), *Npy* (Mm01410146_m1), *Nr2f2* (Mm00772789_m1), *Nrgn* (Mm01178296_g1), *Nts* (Mm00481140_m1), *Pmch* (Mm01242886_g1), *Pou3f3* (Mm00843792_s1), *Pvalb* (Mm00443100_m1), *Reln* (Mm00465200_m1), *Snord116* (Mm05911478_g1), *Sst* (Mm00436671_m1), *Stim2* (Mm01223103_m1), *Tac1* (Mm01166996_m1), *Tac2* (Mm01160362_m1), *Vglut1* (Mm00812886_m1), *Vglut2* (Mm00499876_m1). Target amplification was performed using the CFX Opus Real-Time PCR System (384 well, Bio-Rad) with Bio-Rad CF Maestro 1.1 software version 4.1.

### Quantification and statistical analysis

No statistical methods were used to pre-determine sample sizes, but our sample sizes are similar to those reported in previous publications^9,39,51^. Prism version 9.5.1 was used for statistical analysis. Data collection and processing were randomized or performed in a counterbalanced manner. In the figures, NS indicates *P* ≥ 0.05, *P < 0.05, **P < 0.01, ***P < 0.001, ****P < 0.0001. Exact *P* values are given in the figure legends. Data distribution was assumed to be normal, but this was not formally tested. For nesting behavior, NREM and REM sleep and EEG power spectrum analysis, two-way RM ANOVA with Bonferroni correction and the mixed-effects model were used. For before–after comparisons, the non-parametric two-tailed Wilcoxon matched-paired signed-rank test was used. For sleep architecture analysis and nest scores, the non-parametric two-sided Mann–Whitney *U*-test was used. For electrophysiology, the non-parametric two-tailed Wilcoxon matched-paired signed-rank test was used. Mice were excluded from the analysis if the histology did not confirm AAV transgene expression in the PFC or the VC. While experimenters were not blinded to treatments, data analysis was carried out blindly.

### Reporting summary

Further information on research design is available in the Nature Portfolio Reporting Summary linked to this article.

## Online content

Any methods, additional references, Nature Portfolio reporting summaries, source data, extended data, supplementary information, acknowledgements, peer review information; details of author contributions and competing interests; and statements of data and code availability are available at 10.1038/s41593-023-01430-4.

## Supplementary information

Supplementary Information Supplementary Tables 1 and 2.

Reporting Summary

## Data Availability

The preprocessed raw data can be accessed at Zenodo 8278973.

## Code availability

MATLAB scripts for photometry and EEG power analysis have been deposited at Zenodo 8287112 and 8287079.
