# Author Correction: Somatostatin neurons in prefrontal cortex initiate sleep-preparatory behavior and sleep via the preoptic and lateral hypothalamus

**DOI:** 10.1038/s41593-025-02003-3

**Published:** 2025-06-05

**Authors:** Kyoko Tossell, Xiao Yu, Panagiotis Giannos, Berta Anuncibay Soto, Mathieu Nollet, Raquel Yustos, Giulia Miracca, Mikal Vicente, Andawei Miao, Bryan Hsieh, Ying Ma, Alexei L. Vyssotski, Tim Constandinou, Nicholas P. Franks, William Wisden

**Affiliations:** 1 Department of Life Sciences, Imperial College London, London, UK; 2 Center for Excellence in Brain Science and Intelligence Technology, Chinese Academy of Sciences, Shanghai, China; 3 UK Dementia Research Institute, Imperial College London, London, UK; 4 Department of Electrical and Electronic Engineering, Imperial College London, London, UK; 5 Center for Neurotechnology, Imperial College London, London, UK; 6 Institute of Neuroinformatics, University of Zürich–ETH Zürich, Zürich, Switzerland; 7 Care Research and Technology Centre, UK Dementia Research Institute, London, UK

**Keywords:** Non-REM sleep, Physiology

Correction to: *Nature Neuroscience* 10.1038/s41593-023-01430-4, published online 21 September 2023.

In the version of this article initially published, due to a figure preparation error, in Extended Data Fig. 3b and c (on Dox and off Dox) an incorrect DAPI background was used in the images, which are now updated in the HTML and PDF versions of the article. For comparison, the original and revised images are available in the Supplementary Information accompanying this amendment.

## Supplementary information

Original and revised Extended Data Fig 3

