# Supplementary material for: Somatostatin neurons in prefrontal cortex initiate sleep-preparatory behavior and sleep via the preoptic and lateral hypothalamus
Source: Nat Neurosci. 2023 Sep 21;26(10):1805–19. doi: 10.1038/s41593-023-01430-4 (PMC10545541; doi:10.1038/s41593-023-01430-4)
Supplement: Supplementary file 1 — Supplementary Tables 1 and 2. [file 41593_2023_1430_MOESM1_ESM.pdf]

Table S1. Animal details.

Animal names	Animal lines	Injection location	Virus	Optic-fibre implant site
Vgat-PFC-ChR2-Tag	*Vgat-Cre*	PFC	AAV1/2-cFos-tTA<br>AAV1/2-$P_{TRE\text{-}tight}$-flex-ChR2-EYFP	PFC (right or left)
Vgat-VC-ChR2-Tag	*Vgat-Cre*	VC	AAV1/2-cFos-tTA<br>AAV1/2-$P_{TRE\text{-}tight}$-flex-ChR2-EYFP	VC (right or left)
Vgat-PFC-YFP	*Vgat-Cre*	PFC	AAV1/2-flex-EYFP	PFC (right or left)
Vgat-PFC-hM3Dq-Tag	*Vgat-Cre*	PFC	AAV1/2-cFos-tTA<br>AAV1/2-$P_{TRE\text{-}tight}$-flex-ChR2-EYFP	n/a
Sst-PFC-hM3Dq-Tag	*Sst-Cre*	PFC	AAV1/2-cFos-tTA-pA<br>AAV1/2-$P_{TRE\text{-}tight}$-flex-ChR2-EYFP	n/a
Nos1-PFC-hM3Dq-Tag	*Nos1-Cre*	PFC	AAV1/2-cFos-tTA<br>AAV1/2-$P_{TRE\text{-}tight}$-flex-ChR2-EYFP	n/a
Sst-PFC-ChR2	*Sst-Cre*	PFC	AAV1/2-EF1α-flex-hChR2(H314R)-EYFP<br>or<br>AAV1/2-EF1α-flex-hChR2(H314R)-mCherry	PFC/LPO/LH<br>(right or left)
Sst-PFC-ChR2-Tag	*Sst-Cre*	PFC	AAV1/2-cFos-tTA<br>AAV1/2-$P_{TRE\text{-}tight}$-flex-ChR2-EYFP<br>or<br>AAV1/2-cFos-tTA<br>AAV1/2-$P_{TRE\text{-}tight}$-flex-ChR2-mCherry	PFC/LPO/LH<br>(right or left)
Sst-PFC-GCaMP6-Tag	*Sst-Cre*	PFC	AAV1/2-cFos-tTA<br>AAV1/2-$P_{TRE\text{-}tight}$-flex-GCaMP6-EYFP	LPO/LH<br>(right or left)

Figures	Abbreviation	Animal	Condition
Fig.1<br>ED.Fig.2a-c		*Vgat-Cre*	
Fig.2<br>ED.Fig.4	PFC	*Vgat-PFC-ChR2-Tag:SD*	Activity-tagged
	VC	*Vgat-VC-ChR2-Tag:SD*	
	Ctrl	*Vgat-PFC-ChR2-Tag:Ctrl*<br>or<br>*Vgat-PFC-YFP*	Off-Dox with no tagging
ED.Fig.2d		*Vgat-PFC-YFP::Vgat-PFC-hM3Dq-Tag:SD*	Activity-tagged
		Vgat-VC-YFP::Vgat-VC-hM3Dq-Tag:SD	Activity-tagged
ED.Fig.3		*Vgat-PFC-hM3Dq-Tag:SD*	a: SD:off Dox<br>b: SD:on Dox<br>c: SD:on Dox<br>d: no SD: off Dox
Fig.3<br>ED.Fig.5a,c-e		*Vgat-PFC-hM3Dq-Tag:SD*	Activity-tagged
		Sst-PFC-hM3Dq-Tag:SD	
		Nos1-PFC-hM3Dq-Tag:SD	
ED.Fig.5b		*Vgat-PFC-ChR2-Tag:SD*	Activity-tagged
Fig.4<br>ED.Fig.6b-d	Tag:Ctrl	*Sst-PFC-ChR2-Tag:Ctrl*<br>:same animal (paired) to Tag:SD	On-Dox with no tagging
	Tag:SD	*Sst-PFC-ChR2-Tag:SD*<br>:same animal (paired) to Tag:Ctrl	Activity-tagged
ED.Fig.6a		*Sst-PFC-YFP::Sst-PFC-hM3Dq-Tag:SD*	Activity-tagged
Fig.5<br>ED.Fig.7	PFC^{Sst} cells	*Sst-PFC-ChR2*	
	$PFC^{Sst\text{-}Tag:SD}$ cells	*Sst-PFC-ChR2-Tag:SD*	Activity-tagged
ED.Fig.8a		*Sst-PFC-ChR2-Tag:SD*	Activity-tagged
ED.Fig.8b		*Sst-PFC-ChR2*	
		Sst-PFC-ChR2-Tag:SD	Activity-tagged
ED.Fig.8c		*Sst-VC-ChR2*	
Fig.6<br>Fig.7<br>ED.Fig.9a-e	No light	*Sst-PFC-ChR2,* optic fibre in LPO	No light stimulation
	Light	*Sst-PFC-ChR2,* optic fiber in LPO	5mW stimulation
	Tag:Ctrl	*Sst-PFC-ChR2-Tag:Ctrl,* optic fibre in LPO<br>:same animal (paired) to Tag:SD	On-Dox with no tagging
	Tag:SD	*Sst-PFC-ChR2-Tag:SD,* optic fibre in LPO<br>:same animal (paired) to Tag:Ctrl	Activity-tagged
ED.Fig.9f		*Sst-PFC-GCaMP6-Tag:SD,* optic fibre in LPO	Activity-tagged
Fig.8<br>ED.Fig.10a-c	No light	*Sst-PFC-ChR2,* optic fibre in LH	No light stimulation
	Light	*Sst-PFC-ChR2,* optic fiber in LH	5mW stimulation
	Tag:Ctrl	*Sst-PFC-ChR2-Tag:Ctrl,* optic fibre in LH<br>:same animal (paired) to Tag:SD	On-Dox with no tagging
	Tag:SD	*Sst-PFC-ChR2-Tag:SD,* optic fibre in LH<br>:same animal (paired) to Tag:Ctrl	Activity-tagged
ED. Fig.10d	*Sst-PFC-GCaMP6-Tag:SD*	*Sst-PFC-GCaMP6-Tag:SD,* optic fibre in LH	Activity-tagged

Table S2. Virus volumes and concentrations used for experiments.

(vg = viral genomes)

Figures	Virus	Source	Stock titre
Fig.2, 4, 6, 7, 8<br>ED.Fig.4<br>ED.Fig.5b<br>ED.Fig.6b-d<br>ED.Fig.9a-e<br>ED.Fig.10a-c	Per injection:<br>• 0.125µL *AAV1/2-cFos-tTA*<br>• 0.125µL *AAV1/2-P$_{TRE\text{-}tight}$-flex-ChR2-EYFP*	In-house	 1.2e7 vg/µL<br>6.2e5 vg/µL
Fig.2	Per injection:<br>• 0.125µL *AAV1/2-hsyn-flex-EYFP*<br>• 0.125µLSaline	In-house	1.4e7 vg/µL
Fig.6a-f<br>Fig.8a-d<br>ED.Fig.4b-f<br>ED.Fig.9a-d<br>ED.Fig.10a-b	Per injection:<br>• 0.125µL *AAV1/2-EF1α-flex-hChR2(H314R)-EYFP*<br>• 0.125µL Saline	In-house	 4.6e6 vg/µL
Fig.4<br>ED.Fig.3<br>ED.Fig.5a&c-e	Per injection:<br>• 0.125µL *AAV1/2-cFos-tTA*<br>• 0.125µL *AAV1/2-P$_{TRE\text{-}tight}$-flex-hM3Dq-mCherry*	In-house	 1.2e7 vg/µL<br>3.9e6 vg/µL
Fig.5a-d<br>ED.Fig.7	Per injection:<br>• 0.125µL *AAV1/2-cFos-tTA*<br>• 0.125µL *AAV1/2-P$_{TRE\text{-}tight}$-flex-ChR2-mCherry*	In-house	 1.2e7 vg/µL<br>7.3e5 vg/µL
Fig.5a-d<br>ED.Fig.7	Per injection:<br>• 0.125µL *AAV1/2-EF1α-flex-hChR2(H314R)-mCherry*<br>• 0.125µL Saline	In-house	 1.4e6 vg/µL
ED.Fig.9f<br>ED.Fig.10d	Per injection:<br>• 0.125µL *AAV1/2-cFos-tTA*<br>• 0.125µL *AAV1/2-P$_{TRE\text{-}tight}$-flex-GCaMP6 -EYFP*	In-house	 1.2e7 vg/µL<br>1.9e6 vg/µL
ED.Fig.2d<br>ED.Fig.6a	Per injection:<br>• 0.14µL *AAV1/2-cFos-tTA*<br>• 0.14µL *AAV1/2-P$_{TRE\text{-}tight}$-flex-hM3Dq-mCherry*<br>• 0.07µL *AAV1/2-hsyn-flex-EYFP*	In-house	 1.2e7 vg/µL<br>1.6e5 vg/µL<br>1.4e7 vg/µL
ED.Fig.8a,b	Per injection:<br>• 0.175µL *AAV1/2-cFos-tTA*<br>• 0.175µL *AAV1/2-P$_{TRE\text{-}tight}$-flex-ChR2-EYFP*	In-house	 1.2e7 vg/µL<br>6.2e5 vg/µL
Fig.5e,<br>ED.Fig.8b,c	Per injection:<br>• 0.175µL *AAV1/2-EF1α-flex-hChR2(H314R)-EYFP*<br>• 0.175µL Saline	In-house	 4.6e6 vg/µL
