# Supplementary material for: Somatostatin neurons in prefrontal cortex initiate sleep-preparatory behavior and sleep via the preoptic and lateral hypothalamus
Source: Nat Neurosci. 2023 Sep 21;26(10):1805–19. doi: 10.1038/s41593-023-01430-4 (PMC10545541; doi:10.1038/s41593-023-01430-4)
Supplement: Supplementary file 2 — Reporting Summary [file 41593_2023_1430_MOESM2_ESM.pdf]

nature research

Corresponding author(s): Nicholas P. Franks, William Wisden

Last updated by author(s): 14 August 2023

Reporting Summary

Nature Research wishes to improve the reproducibility of the work that we publish. This form provides structure for consistency and transparency in reporting. For further information on Nature Research policies, see our Editorial Policies and the Editorial Policy Checklist.

Statistics

For all statistical analyses, confirm that the following items are present in the figure legend, table legend, main text, or Methods section.

n/a	Confirmed	
☐	☒	The exact sample size (*n*) for each experimental group/condition, given as a discrete number and unit of measurement
☐	☒	A statement on whether measurements were taken from distinct samples or whether the same sample was measured repeatedly
☐	☒	The statistical test(s) used AND whether they are one- or two-sided<br>*Only common tests should be described solely by name; describe more complex techniques in the Methods section.*
☐	☒	A description of all covariates tested
☐	☒	A description of any assumptions or corrections, such as tests of normality and adjustment for multiple comparisons
☐	☒	A full description of the statistical parameters including central tendency (e.g. means) or other basic estimates (e.g. regression coefficient) AND variation (e.g. standard deviation) or associated estimates of uncertainty (e.g. confidence intervals)
☐	☒	For null hypothesis testing, the test statistic (e.g. *F*, *t*, *r*) with confidence intervals, effect sizes, degrees of freedom and *P* value noted<br>*Give P values as exact values whenever suitable.*
☒	☐	For Bayesian analysis, information on the choice of priors and Markov chain Monte Carlo settings
☒	☐	For hierarchical and complex designs, identification of the appropriate level for tests and full reporting of outcomes
☒	☐	Estimates of effect sizes (e.g. Cohen's *d*, Pearson's *r*), indicating how they were calculated

Our web collection on statistics for biologists contains articles on many of the points above.

Software and code

Policy information about availability of computer code

Data collection	EEG/EMG signals were recorded using Neurologger 2A devices (Anisimov et al., Nat Methods 2014). Behavior was recorded by video camera which was placed above the test cage. Core body temperature was recorded using temperature loggers (DST nano, Star-Oddi) implanted abdominally. Electrophysiology data was recorded using Multiclamp700B amplifier, Digidata 1440A interface, and Clampfit v10.7 software (Molecular Devices). Target RT-PCR amplification was performed by using CFX Opus Real-Time PCR system (384-well, Bio-Rad).Calcium photometry signals were recorded using Spike2 software (Cambridge Electronic Design) or Doric Neuroscience Studio v6 (Doric Lenses)
Data analysis	Spike 2 software v. 7.20 (Cambridge Electronic Design, Cambridge, UK) was used for sleep scoring. The open source software Behavioural Observation Research Interactive Software v. 7.8.2 (BORIS) was used for behaviour analysis. Custom-made code in Matlab v2018a and v2019a (The MathWorks Inc, version v2018a) was used for aligning sleep, behavior scores, body temperature and photometry signals. mIPSCs were analysed with MiniAnalysis (Synaptosoft). Bio-Rad CF Maestro 1.1 software v4.1 (Bio-Rad) was used for Cq value analysis. GraphPad Prism v 9.5.1. (GraphPad Software, San Diego, USA) was used for statistical analysis. Fiji v. 2.9.0 was used to merge fluorescent images and add scale bars. Figures were prepared in Adobe Illustrator v27.5.

For manuscripts utilizing custom algorithms or software that are central to the research but not yet described in published literature, software must be made available to editors and reviewers. We strongly encourage code deposition in a community repository (e.g. GitHub). See the Nature Research guidelines for submitting code & software for further information.

Data

Policy information about availability of data

All manuscripts must include a data availability statement. This statement should provide the following information, where applicable:

- Accession codes, unique identifiers, or web links for publicly available datasets
- A list of figures that have associated raw data
- A description of any restrictions on data availability

All raw data and analysis code are available from the corresponding authors on request.

Field-specific reporting

Please select the one below that is the best fit for your research. If you are not sure, read the appropriate sections before making your selection.

☒ Life sciences ☐ Behavioural & social sciences ☐ Ecological, evolutionary & environmental sciences

For a reference copy of the document with all sections, see nature.com/documents/nr-reporting-summary-flat.pdf

Life sciences study design

All studies must disclose on these points even when the disclosure is negative.

Sample size	Sample sizes were initially based on previous studies using chemogenetics and optogenetic tools for the studies of sleep-wake circuitry.
Data exclusions	Any activity-tagged datasets which did not have AAV expression in the injected area were excluded prior to the analysis in post hoc. Animals did not displayed standard sleep/wake patterns in EEG/EMG during random 24hrs baseline recording (up to 3 times) were excluded from activity-tagging process.
Replication	Multiple mice from independent cohorts were used. The numbers of animals, recording sessions, opto-stimulation trials, or cells were indicated on the figure legends. Histology images shows in Figures were repeated in at least three mice.
Randomization	Selection of animals and the sequence of experimental sessions were randomized.
Blinding	The experimenters could not successfully be blinded to the genotype of the transgenic animals during sleep scoring and behavior scoring because the characteristic features of increased amount of NREM/REM sleep became apparent during the scoring process. Nesting behaviour and nest scores were carried out by multiple experimenters separately, and main experimenter's scores were used only when overall difference between experimenters result were less than 5%.

Reporting for specific materials, systems and methods

We require information from authors about some types of materials, experimental systems and methods used in many studies. Here, indicate whether each material, system or method listed is relevant to your study. If you are not sure if a list item applies to your research, read the appropriate section before selecting a response.

Materials & experimental systems

n/a	Involved in the study	
☐	☒	Antibodies
☐	☒	Eukaryotic cell lines
☒	☐	Palaeontology and archaeology
☐	☒	Animals and other organisms
☒	☐	Human research participants
☒	☐	Clinical data
☒	☐	Dual use research of concern

Methods

n/a	Involved in the study	
☒	☐	ChIP-seq
☒	☐	Flow cytometry
☒	☐	MRI-based neuroimaging

Antibodies

Antibodies used	Primary antibodies: rabbit anti-GFP (Invitrogen, A6455, 1:1000), chicken anti-GFP (Abcam, ab13970, 1:1000), mouse-anti-mCherry (Clontech, 632543 1:1000); rabbit polyclonal cFOS (Santa Cruz Biotechnology, sc-52, 1:4000); mouse monoclonal Gad67, (Millipore, MAB5406, 1:500).<br>Secondary antibodies:Alexa Fluor-488 goat anti-chicken (Invitrogen, A11039), Alexa Fluor-488 goat anti-rabbit (Invitrogen, A11008), Alexa Fluor-594 goat anti-mouse (Invitrogen, A11005), and Alexa Fluor488-conjugated streptavidin (Invitrogen, S1123).
Validation	Primary antibody specificity verification for anti-GFP and anti-mCherry was done on control C57BL6/J brain tissue with non-flexed GFP or mCherry AAV expression. The Gad67 and cFos antibodies were validated by the manufacturers on western blots and by

immunohistochemical analysis. The c-FOS antibody has been used by many investigators and does seem to reflect neuronal activity in its pattern of staining in mouse; it also stains the nucleus of the cell rather than the cytoplasm, as expected of a transcription factor. The Gad 67 antibody gives the expected expression e.g., neocrotical and hippocampal interneurons, reticular thalamus, cerebellar Purkinje cells, Functional application validation was performed by using control brain tissues without appropriate antibodies.

Eukaryotic cell lines

Policy information about cell lines

Cell line source(s)	HEK293 cells, Sigma-Aldrich, 85120602/CVCL_0045
Authentication	The cell line was authenticated. But the cell line was used only to package AAV and not to produce biological data
Mycoplasma contamination	The cell line tested negative for mycoplasma contamination
Commonly misidentified lines (See ICLAC register)	No commonly misidentified cell lines were used

Animals and other organisms

Policy information about studies involving animals; ARRIVE guidelines recommended for reporting animal research

Laboratory animals	Vgat-ires-Cre (Jackson laboratory: stock 016962) mice, Nos1-ires-Cre (Jackson laboratory: stock 017526), Sst-ires-Cre (Jackson laboratory: stock 013044) and C57BL/6J mice, 2-5 month-old male and female mice were used in this study.
Wild animals	No wild animals were used in this study.
Field-collected samples	The study did not involve samples collected from the field.
Ethics oversight	All experiments were performed in accordance with the United Kingdom Animal Scientific Procedures Act 1986 under personal and project licenses granted by the United Kingdom Home Office. Ethical approval was provided by the Ethical Review Panel at the Imperial College London.

Note that full information on the approval of the study protocol must also be provided in the manuscript.
