# Supplementary material for: Author Correction: Somatostatin neurons in prefrontal cortex initiate sleep-preparatory behavior and sleep via the preoptic and lateral hypothalamus
Source: Nat Neurosci. 2025 Jun 5;28(7):1570. doi: 10.1038/s41593-025-02003-3 (PMC12229885; doi:10.1038/s41593-025-02003-3)

nature neuroscience

Supplementary information

Author Correction: Somatostatin neurons in prefrontal cortex initiate sleep-preparatory behavior and sleep via the preoptic and lateral hypothalamus

In the format provided by the authors and unedited

Original Extended Data Fig. 3

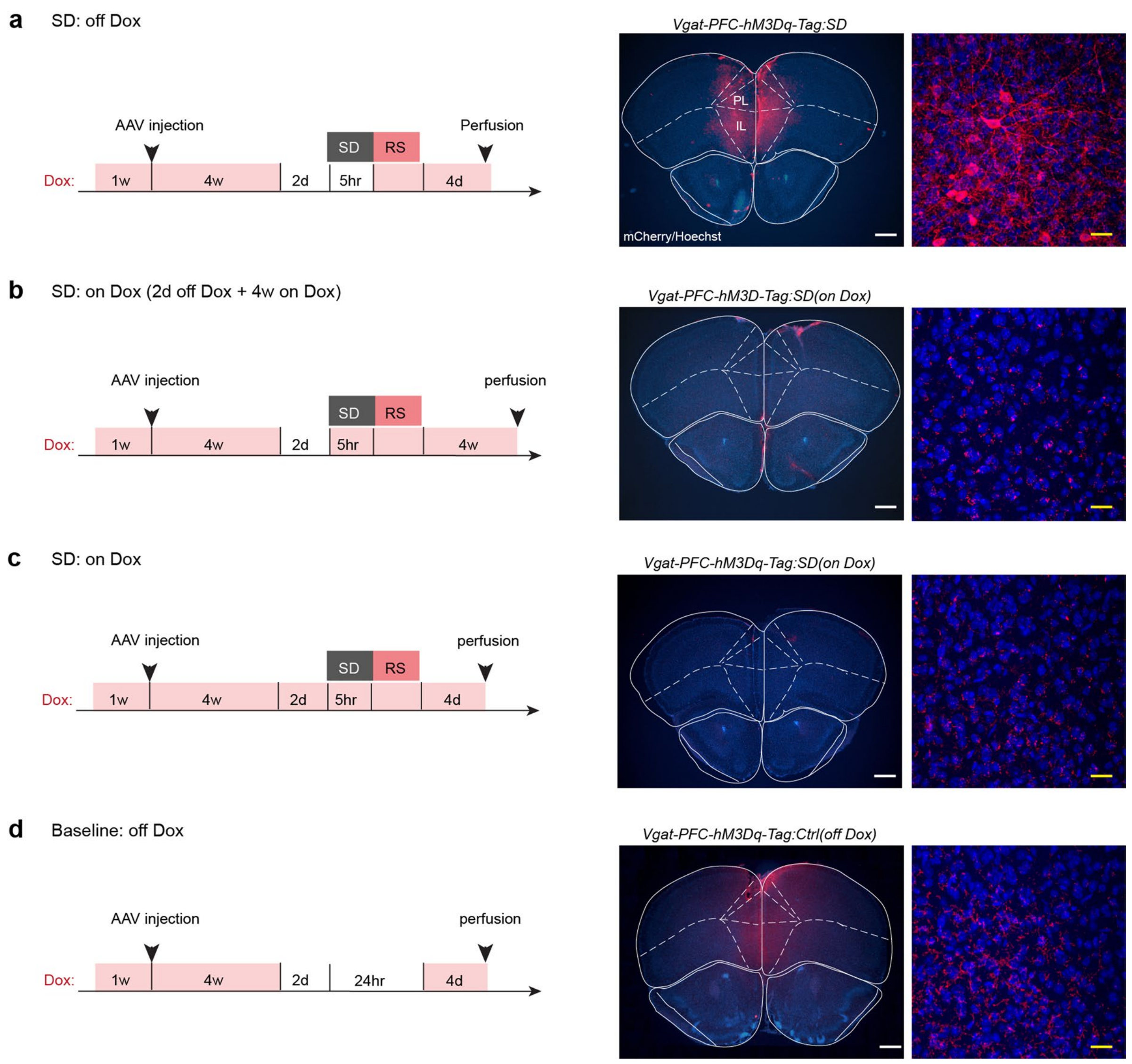

Updated Extended Data Fig. 3

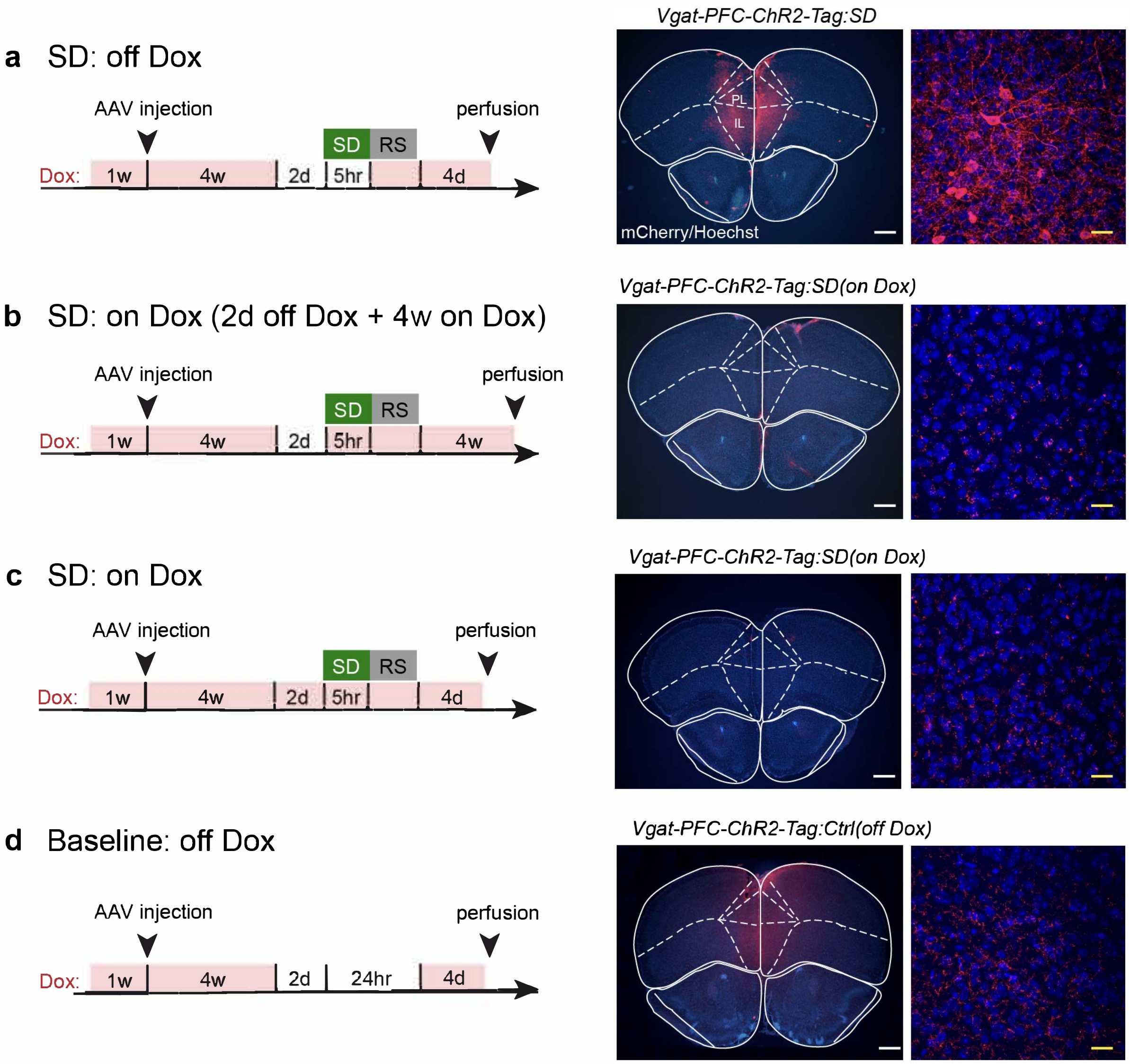

Supplement: Supplementary file 1 — Original and revised Extended Data Fig 3 [file 41593_2025_2003_MOESM1_ESM.pdf]
